# An automated iterative approach for protein structure refinement using pseudocontact shifts

**DOI:** 10.1007/s10858-021-00376-8

**Published:** 2021-08-02

**Authors:** Stefano Cucuzza, Peter Güntert, Andreas Plückthun, Oliver Zerbe

**Affiliations:** 1 Department of Chemistry, University of Zürich, Winterthurerstrasse 190, 8057 Zürich, Switzerland; 2 Institute of Biophysical Chemistry and Center for Biomolecular Magnetic Resonance, Goethe University Frankfurt, Max-von-Laue-Straße 9, 60438 Frankfurt am Main, Germany; 3 Laboratory of Physical Chemistry, ETH Zürich, Vladimir-Prelog-Weg 2, 8093 Zürich, Switzerland; 4 Department of Chemistry, Tokyo Metropolitan University, 1–1 Minami-Osawa, Hachioji, 192-0397 Tokyo, Japan; 5 Department of Biochemistry, University of Zürich, Winterthurerstrasse 190, 8057 Zürich, Switzerland

**Keywords:** Pseudocontact shift, NMR, Armadillo repeat protein, Structure refinement

## Abstract

NMR structure calculation using NOE-derived distance restraints requires a considerable number of assignments of both backbone and sidechains resonances, often difficult or impossible to get for large or complex proteins. Pseudocontact shifts (PCSs) also play a well-established role in NMR protein structure calculation, usually to augment existing structural, mostly NOE-derived, information. Existing refinement protocols using PCSs usually either require a sizeable number of sidechain assignments or are complemented by other experimental restraints. Here, we present an automated iterative procedure to perform backbone protein structure refinements requiring only a limited amount of backbone amide PCSs. Already known structural features from a starting homology model, in this case modules of repeat proteins, are framed into a scaffold that is subsequently refined by experimental PCSs. The method produces reliable indicators that can be monitored to judge about the performance. We applied it to a system in which sidechain assignments are hardly possible, designed Armadillo repeat proteins (dArmRPs), and we calculated the solution NMR structure of YM_4_A, a dArmRP containing four sequence-identical internal modules, obtaining high convergence to a single structure. We suggest that this approach is particularly useful when approximate folds are known from other techniques, such as X-ray crystallography, while avoiding inherent artefacts due to, for instance, crystal packing.

**Supplementary Information:**

The online version contains supplementary material available at 10.1007/s10858-021-00376-8.

## Introduction

A currently very active area in biomedical research is the design of proteins with antibody-like properties that circumvent disadvantages of real antibodies (Banta et al. 2013; Binz et al. 2005; Jost and Plückthun 2014). Natural antibodies achieve high affinity by randomizing epitopes and efficiently selecting for best binders. The rational design of proteins mimicking such interactions unfortunately proved to be very difficult. Part of the problem is that, despite intense research, it is still almost impossible to predict the structure of good binders solely based on the target sequence. We are trying to bypass this problem by creating binding modules optimized by directed evolution to bind specific dipeptide sequences in the context of a longer peptide, which can then be assembled in the required order *in silico* into designed Armadillo repeat proteins (dArmRPs) to create a binder (Reichen et al. 2014a). The approach has the advantage of potentially reducing the problem of finding a high affinity binder for each new sequence to the problem of finding a binding module for a dipeptide. We anticipate that such a rational approach to designing binders would bring massive advantages in the fields of research, diagnostics and therapeutics (Jost and Plückthun 2014; Simeon and Chen 2018).

**Fig. 1:**
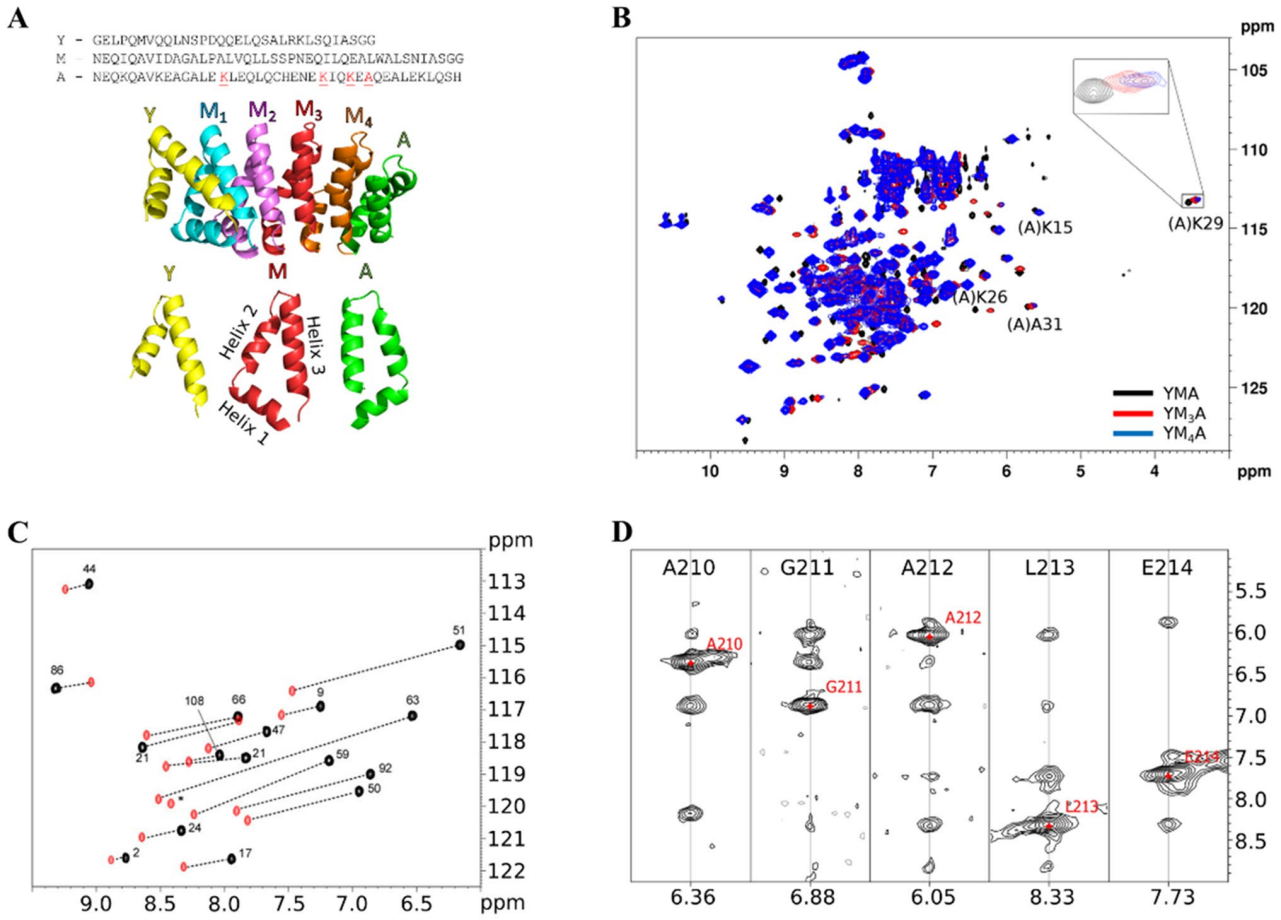
Assignment of YM_4_A. **a** YMA sequence (top), in proteins with more internal repeats, the M sequence would be repeated; cartoon representation of YM_4_A (center); details of the two caps Y and A and an internal M module (bottom). **b** Superposition of [^15^N,^1^H]-HSQC spectra of (A)S21C YM_*n*_A tagged with Tm-4R4S-DOTA-M8, where n = 1, 3, 4 in black, red and blue, respectively. Signals corresponding to residues in the C-terminal A cap are annotated in the spectra and underlined in the sequence. **c** Overlay of [^15^N,^1^H]-HSQC spectra of single ^15^N-Leu-labeled (A)S21C YMA, coupled to the paramagnetic tag (in black), or untagged (in red). The induced PCS are depicted by a dashed line. The paramagnetic partner of the peak with the star is located outside the displayed range. **d** Strips of the ^15^N-resolved NOESY displaying correlations of amide protons for residues 210–214 of (M3)S21C YM_4_A tagged with Tm-3R4S-DOTA-M7 thiazole, highlighting the use of an unambiguous anchor assignment identified from the ^15^N-Leu sample, L213, as starting point to obtain neighbors assignments

These dArmRPs are synthetic homologs derived from a family of naturally occurring α-solenoid repeat proteins, natural Armadillo repeat proteins (nArmRPs), which are able to bind small stretches of unstructured peptides or proteins (Conti et al. 1998; Conti and Kuriyan 2000; Huber and Weis 2001). They form elongated, rod-like molecules that consist of multiple, tightly packed internal modules M, and are terminated at the N- and C-terminal ends by capping modules Y and A, respectively (Fig. 1a). Each internal module M contains three tightly packed α-helices H_1_, H_2_, and H_3_. They propagate a right-handed triangular spiral, which exposes a supercoiled binding surface consisting of helix H_3_ of each repeat (Michel et al. 2018). In view of their potential role as antibody substitutes, they display the favorable feature of binding unstructured peptides, as demonstrated for neurotensin (Ewald et al. 2015; Varadamsetty et al. 2012) or peptides comprising lysine-arginine (KR) dipeptide repeats (Hansen et al. 2017; Reichen et al. 2016b). In the latter case, X-ray structures confirmed that the bound peptide was in an extended conformation, and the interactions corresponded to those of the natural ArmRPs, in an extended and idealized way.

The above-described modular approach of creating binders using repeat proteins requires high affinity and selectivity of the individual modules. It poses the significant challenge of selecting a scaffold that allows to combine different modules such that overall geometric features required for modular binding are retained. Here, structural biology plays a pivotal role in order to determine the overall molecular features with high precision. In particular, changes in the supercoil of the scaffold will influence distances between atoms of the ligand and atoms of the binder. High-resolution crystal structures of several dArmRPs have been determined over the years, confirming the sequence-specific binding of dArmRPs to different target peptides (Hansen et al. 2016, 2018; Reichen et al. 2014b, 2016a). However, the intrinsic α-solenoid structures crystallize in a tubular arrangement, which in turn potentially influences packing and curvature of the dArmRPs (Hansen et al. 2018). Other artefacts due to crystal forces were also observed, such as register shifts for the bound peptides, steric clashes leading to displacements or multiple binding partners (Reichen et al. 2014b, 2016b).

Solution nuclear magnetic resonance (NMR) spectroscopy in principle presents a highly valuable complementary technique to crystallography, because it determines the structure in the absence of packing forces. However, the repetitive nature of dArmRPs causes a significant assignment problem and is also often accompanied by resonance overlap of signals from identical positions in the corresponding repeats. With substantial efforts, our group managed to fully assign the protein backbone by using a number of different approaches, mostly based on segmental labelling (manuscript in preparation). However, sidechain assignments were not possible, and hence critical distance restraints to determine a high-resolution structure from NOE data are not yet available (Wagner and Wüthrich 1982). Even if those NOEs could be assigned, error propagation along a network of NOEs would not allow to compute structures in which remote parts can be aligned with the required high accuracy. In principle, both residual dipolar couplings (RDCs) (Bax et al. 2001; Tjandra and Bax 1997) and pseudo-contact shifts (PCSs) (Bertini et al. 2002; Koehler and Meiler 2011; Nitsche and Otting 2017; Otting 2010) allow to orient position of atoms relative to a common frame, and hence can establish relative orientations of remote units with high reliability. In order to use RDCs for structure refinement, a large set of RDCs must be measured, however, some of which are difficult to derive experimentally with sufficient precision for large proteins.

PCSs can be induced by coupling lanthanide-chelating tags to Cys residues (Barthelmes et al. 2011; Clore and Iwahara 2009; Joss and Häussinger 2019; Keizers and Ubbink 2011) and they provide long-range structural information, which is both distance- and orientation-dependent (Bertini et al. 2002; Koehler and Meiler 2011; Parigi et al. 2019). So far, PCSs have mostly been used to refine approximately known structures or to orient domains relative to each other. In order to determine the *Δχ-*tensor accurately a considerable number of backbone assignments are required to extract a sufficient number of PCSs as well as a reasonably close starting structure. Unfortunately, both requirements are not met in the case of dArmRPs, as described below.

PCS refinement of repeat proteins, therefore, faces two major challenges: (i) repeat proteins are difficult to fully assign due to the repetitive nature of the amino acid sequence, and hence only a limited set of PCS data is available. (ii) high precision and accuracy are required so that data useful for design purposes can be generated. Much of what would usually be considered a high-quality structure is insufficiently accurate in our case.

Herein we develop, test and apply a refinement protocol to refine structures of repeat proteins based on an incomplete set of experimental backbone PCSs. The PCSs are applied to refine a scaffold extracted from a homology model. We demonstrate that this protocol can compute accurate structures even in the presence of only a limited number of assignments. Confidence in the correctness of the protocol is provided by several indicators that can be monitored during the calculation to help in identifying errors either in the structure calculation procedure or in the assignments.

In order to obtain as many reliable PCSs as possible, we exploit the modular nature of dArmRPs to facilitate the assignment procedure. We then demonstrate that it is possible to re-calculate a structure from scaffold restraints and a limited amount of experimental PCSs. Subsequently, we develop an iterative procedure in which the target structure is computed from a distorted structure. In each iteration, scaffold restraints and *Δχ-*tensor components are updated based on the structure obtained from the previous cycle. We also tested convergence and robustness of the procedure by starting from differently distorted structures and investigated how convergence is influenced by a number of parameters of the refinement protocol. Finally, we applied the protocol to compute the solution NMR structure of a dArmRP, YM_4_A, based on a model that was refined with high accuracy and precision using exclusively backbone PCSs.

## Materials and methods

### Cloning and mutagenesis

YMA and YM_4_A genes were cloned into the vector pEM3BT2 (Michel et al. 2018) containing a TEV-cleavable N-terminal (His)_6_-GB1 domain (Michel and Wüthrich 2012) using XbaI and BamHI restriction sites. Subsequently, Cys mutants were generated using the QuikChange II mutagenesis protocol (Stratagene) utilizing primers purchased from Microsynth and reported in the SI.

### Protein expression and purification

Unlabeled or uniformly labeled proteins were expressed and purified according to a previously described protocol (Michel et al. 2019). To produce amino acid-specific isotope-labelled protein, precultures were inoculated in NH_4_Cl-free M9 medium complemented with 19 unlabeled amino acids (Sigma) at 37 °C for 16 h and then transferred into 1 L M9 at 37 °C until OD600 of 0.6, at which point the ^15^N-labelled amino acid was introduced and expression was induced with 0.1 mM IPTG at 30 °C for 16 h (SI).

### Site-specific spin labelling

Tm-4R4S-DOTA-M8 and Tm-3R4S-DOTA-M7Thiazole tags for producing paramagnetic proteins (Thulium) and their corresponding diamagnetic reference (Lutetium) were provided by the group of Prof. Dr. D. Häussinger and attached to sidechains of uniquely introduced Cys residues according to a previously described protocol (Müntener et al. 2018). In brief, 150 µM of protein solution in tagging buffer (20 mM Na_2_HPO_4_, 0.2 mM TCEP, pH 7.0) was separated from the reducing agent using a PD-10 column (Sigma) and immediately incubated with a fivefold excess of lanthanide tag overnight at room temperature shaking at 300 rpm.

### NMR measurements

Tagged proteins were buffer-exchanged to NMR buffer (20 mM Na_2_HPO_4_, 2 mM trimethylsilylpropanoate (TMSP), pH 7.0 and 10 % D_2_O) using ultra centrifugal filters (Amicon) to remove any unreacted lanthanide tag. All experiments were recorded on a Bruker Neo 600 MHz spectrometer, using either cryogenically cooled or Prodigy ^1^H,^13^C,^15^N triple-resonance probes. ^15^N,^1^H NMR experiments used pulsed field gradients with coherence selection (Keeler et al. 1994) and the Rance-Palmer method for sensitivity enhancement (Palmer et al. 1991). To assign the paramagnetic state, we additionally used amide-amide NOEs from a 200 ms NOESY-[^15^N,^1^H]-HSQC experiment. All experiments used standard Bruker pulse sequences. Spectral widths were 15 and 40 ppm in the direct and indirect dimensions, using 1024 or 128 complex data points. All experiments to measure pseudocontact shifts were recorded at 293 K, and adapted to previously determined chemical shifts at 310 K through a series of [^15^N,^1^H]-HSQC spectra recorded in steps of 2 degrees. Protein concentrations of samples were usually in the range of 100–150 µM for tagged and 350–400 µM for untagged samples. Proton chemical shifts were referenced to internal TMSP, from which the ^15^N chemical shifts were indirectly referenced to the liquid ammonia scale using the conversion factor of 0.10132900 (Live et al. 1984). Spectra were processed in TOPSPIN using cosine-bell-shifted window functions prior to Fourier transformation, and analyzed in CARA (Keller 2004).

### PCS tensor fitting

Paramagnetic anisotropic susceptibility tensors (*Δχ-*tensors) and the corresponding back-calculated ^1^H and ^15^N PCS values were calculated with the software Numbat (Schmitz et al. 2008) in case of the non-iterative procedure and with the Python module Paramagpy (Orton et al. 2020) in case of the iterative procedure. An in-house custom modification of Paramagpy was used for the iterative procedure in CYANA, as detailed in the SI and available upon request.

Virtual ^1^H and ^15^N amide backbone PCS values of existing attachment sites, employed during optimizations of procedures and parameter, were generated by calculating the *Δχ-*tensor from the fit of experimental PCSs to the input structure, and then replacing experimental with back-calculated PCSs, thereby obtaining a perfect match between *Δχ-*tensor, PCS values and input structure. When simulating data from virtual attachment sites, a template *Δχ-*tensor, based on the experimental (M3)S21C attachment site fit to the model I structure (Table S14), was applied to a set of *x*, *y*, *z* coordinates representing a realistic position for the virtual site.

### Creation of model structures

The main model structure of YM_4_A (structure A), used for initial assignments and in the non-iterative procedure, was created by deleting the C-terminal A cap from the crystal structures of YM_5_A (PDB ID: 5MFN) and mutating the fifth module into a new A cap through PyMOL’s mutagenesis best rotamers approach. The YMA model was produced in a similar way by deleting three more modules. Additional YM_4_A models for the iterative-procedure, structures B, C and D, were generated by twisting structure A at the M_1_-M_2_ junction, M_3_-M_4_ junction or randomly at every junction, respectively (Fig S1). For the experimental structure refinement of YM_4_A, in addition to structure A (in this context called model I), three additional input structures were generated, model II-IV. Model II was adapted from model I to include a partially unpacked Y cap; model III was derived from a crystal structure of YM_4_A with a new, improved Y cap; model IV was obtained from an unpublished crystal structure of YM_6_A fused at both termini with DARPins (PDB ID: 6SA8).

### Structure refinements

Structure refinement in the non-iterative procedure was performed with the software CYANA (Güntert 2004) version 3.98.12. In order to extract scaffold restraints, the model structure was loaded and upper distance limits (UPL) were generated through the *distances make* command by extracting all distances between atoms within the same module in a 2.5–5.0 Å range with an added 0.5 Å tolerance. Backbone φ and ψ dihedral angles were determined from the structure and added as dihedral angle restraints (ACO) with an additional ± 5° tolerance. Metal tag position (ORI) restraints were generated by calculating the distance between the tag position predicted by the *Δχ-*tensor and six nearby Cα atoms with a custom PyMOL script, and converted into UPL and lower limit (LOL) restraints while adding a tolerance of 0.5 Å. ^1^H and ^15^N PCS values and respective *Δχ-*tensor axial and rhombic components were converted from the Numbat/Paramagpy .npc format to the CYANA .pcs format with a Python script. It has been shown (John et al. 2005) that ^15^N chemical shift anisotropy (CSA) could distort ^15^N PCS values. In order to estimate the impact of ^15^N CSA contributions to ^15^N PCSs, test calculations using solely ^1^H PCS data were performed, which revealed only minor differences (Fig. S17). In general, the ^1^H and ^15^N PCS values (in ppm) of an amide group are expected to be nearly equal because the two atoms are spatially close. Therefore, PCS restraints from amide groups for which the ^1^H and ^15^N PCS values differed by more than 20 % were discarded, possibly removing peaks with substantial ^15^N CSA contributions.

1000–5000 structures were calculated in 10000–100000 MD steps supplying the above restraints to the *calc_all* macro with fixed seed and default weights (UPL/LOL/PCS = 1.0; VdW = 2.0; ACO = 5.0) constant throughout all phases of the simulated annealing with the exception of the VdW weights (0.25, 0.25, 0.25, 1.0). The 10 best conformers ranked by target function value were merged into a bundle for subsequent analysis. To visualize the structural changes during the different phases, the standard CYANA simulated annealing (SA) protocol (Güntert and Buchner 2015) was modified to calculate a single structure in 25000 MD steps and to save a structure snapshot every 200 MD steps, corresponding to 42 snapshots in phase 1 (SA with reduced heavy atom radii; high temperature and initial cooling), 41 snapshots in phase 2 (normal heavy atom radii; continued cooling) and 3 (normal heavy and hydrogen atom radii; continued cooling) and a single snapshot in phase 4 (increased weight for steric repulsion; low temperature). During some of the tests ACO or PCS restraints were switched off by setting the weight in the corresponding phase to 0. Movies from the trajectory were realized in PyMOL and added to the SI.

This procedure was modified in the iterative simulated annealing protocol with a CYANA (Güntert 2004) macro that calls external Python scripts. In a first step, the initial model structure was used to fit a *Δχ-*tensor for each tag with Paramagpy using PCSs derived either from the target structure or experimentally, and the resulting axial and rhombic tensor components were exported in .pcs format together with restraints for the metal tag position (ORI). Subsequently, 5–10 cycles were executed in which: (i) UPL and ACO scaffold restraints were generated by the *regularize* macro (Gottstein et al. 2012); (ii) 500 conformers were calculated with 25000 MD steps supplying the previously determined UPL, ACO, ORI and PCS restraints using the *calc_all* macro with default weights with the exception of strongly increased weights for PCSs (30–50); (iii) the 20–30 best structures ranked by target function value were saved and for each tag a (new) *Δχ-*tensor was fitted to each of them; (iv) out of these 20–30 structures the one with the lowest Q-factor averaged over the three tags, representing the quality of the fit of input PCSs to the computed structure, is saved and used as the input model structure for the next cycle (Fig. 5a).

All structures were visualized in PyMOL and RMSD calculations were performed considering only backbone heavy atoms either with CYANA, the PyMOL *align* function, or custom Python scripts. All calculations were performed on a 16-core cluster with 64 GB RAM.

## Results

In what follows below, we first describe a way to assign PCS for repeat proteins. We developed and then tested a protocol for reproducing a structure from computed PCSs, investigated how this is affected by the availability of only partial assignments, and finally we applied the method to determine the so-far unknown structure of a 25 kDa repeat protein harboring 4 internal sequence-identical repeats.

### Assignment of PCSs

In order to simplify the assignment procedure of YM_4_A, a dArmRP with 4 internal repeats and two capping repeats, we exploited the modular nature of dArmRPs by generating a series of proteins with an increasing number of internal repeats YM_*n*_A with *n* = 1, 2, 3, 4. We started the assignment procedure with the smallest construct YMA. This variant retains the larger construct’s fold, thus enabling us to identify potentially suitable attachment sites for the PCS probe, while rapidly allowing for assignments.

Six potential attachment sites, (Y)S19C, (M)Q5C, (M)D9C, (M)Q18C, (M)S21C and (A)S21C (numbering refers to the individual capping or internal repeats, and the repeat type is indicated in the parenthesis) were tested by introducing the respective Cys mutation and coupling with Lu-4R4S-DOTA-M8 and Tm-4R4S-DOTA-M8. [^15^N,^1^H]-HSQC spectra revealed that (Y)S19C, (M)Q18C, (M)S21C and (A)S21C attach the PCS tag such that it uniquely associates with the protein surface resulting in a single set of peaks with moderate to large pseudocontact shifts (proton PCSs up to 4 ppm). Data from the remaining two sites indicate more flexible tags: (M)Q5C displayed two sets of peaks while (M)D9C displayed very small PCS shifts due to averaging over more than one location, and were therefore discarded (Fig. S2).

In the case of probes attached to (A)S21C and (Y)S19C, the tag is positioned at one of the C- or N-terminal capping repeats, respectively, and would therefore generate additional signals belonging to the extra internal modules in the larger constructs. Importantly, they will also strongly help to identify signals from cap residues themselves, because these possess fairly large PCSs and will appear in all proteins tagged at the same positions at similar places in the spectra. We tested this approach by generating the (A)S21C mutants of YM_2_A, YM_3_A and YM_4_A tagged with Tm-4R4S-DOTA-M8. As expected, several strongly shifted signals appear at similar positions in the spectra that stem from residues at the corresponding positions in the protein (Fig. 1b and Fig. S3.).

Initially, proteins tagged at the four suitable attachment sites were fully assigned for YMA in the diamagnetic state based on the previously obtained assignments of the untagged Cys mutants, which could easily be adapted from those of the wild-type protein. Assignments of the paramagnetic state were performed by obtaining a first set of unambiguous assignments using data from the Tm-4R4S-DOTA-M8 YM_*n*_A series and well-separated isolated regions that were fit to a model YMA structure to produce *Δχ-*tensors. Back-calculated PCSs from the *Δχ-*tensor were then fit to the spectra and validated with amide-amide NOEs until complete sequence assignments were accomplished, with the exception of prolines and residues subject to paramagnetic relaxation enhancement (PRE).

Subsequently, three attachment sites evenly distributed across the protein were selected for YM_4_A: (M_1_)Q18C, (M_3_)S21C and (A)S21C. The site in the N-terminal (Y) capping repeat, despite the promising results on YMA, was excluded due to the apparent partial mobility of the wild-type Y cap. (M_1_)Q18C and (A)S21C were tagged with Tm-4R4S-DOTA-M8 and (M_3_)S21C with the then-available improved version Tm-3R4S-DOTA-M7 Thiazole (Müntener et al. 2018). In order to further facilitate the assignment procedure, we expressed a single ^15^N-Ala- or ^15^N-Leu-labelled sample for each site. These samples contain a small subset of all signals, thus enabling more unambiguous initial assignments which are crucial to obtain a first estimate of the *Δχ-*tensor (Fig. 1c). [^15^N,^1^H]-HSQC spectra of the paramagnetic states of the three YM_4_A attachment sites were then assigned in an iterative fashion. An initial set of around 25 unambiguous assignments was obtained from single amino acid labelling, comparison of spectra from the YM_*n*_A series, sequential amide NOEs (Fig. 1d), and peaks from well-separated isolated regions (Fig. S4).

This initially rather limited set of PCSs was then used to compute a first estimate of the *Δχ-*tensor. Knowledge of the tensor in turn allowed us to predict peak positions based on the chemical shifts of the diamagnetic reference, which were then verified by amide-amide NOEs, and thereby largely helped to obtain more assignments. This procedure was iteratively repeated until no further assignments were possible. In our experience, the commonly used approach of assigning peaks by measuring HSQC spectra at different temperatures in order to interpolate peak positions to those in absence of PCSs proved to be less reliable due to the crowding of the spectra and the uncertainties introduced by the variable temperature drift of peaks, while the rather uncommon approach of using NOESY correlations proved very useful in this case.

Despite the multiple and complementary sources of information in the assignment procedure, the intrinsic complexity resulting from the repetitive sequence and the associated peak overlap enabled us to obtain only a subset of assignments, ranging from 33 to 53 % for the different attachment sites (Table S13). Tensor descriptions and the related error analysis are reported in detail in Supplementary Information (Fig. S5, S6, S7 and Table S14).

### Development of a refinement protocol and tests on a known structure

Since the lack of complete assignments could strongly impact the sampling in the subsequent structure calculation, we set out to create a set of simulations in order to demonstrate that structure calculations do indeed reach the correct structure in the presence of only limited data. The goals of the simulations were multiple: (i) demonstrate that the procedure is able to generate the correct structure when enough data is used (positive control); (ii) prove that the previously obtained assignments are sufficient to generate the correct structure (mapping); (iii) extend our understanding of the simulations and their limitations by probing different conditions.

Our goal at this first stage was not a *de novo* structure determination but rather a refinement, starting from an approximately correct structure. Initial computations using only PCS restraints quickly revealed that the calculations failed to compute the correct fold, a known phenomenon that is due to the fact that solutions to a particular PCS value are not unique (Nitsche and Otting 2017). Since dArmRPs all display similar folds, we decided to individually restrain each module to its canonical geometry, and allow individual modules to reorient with respect to each other. Thereby, PCSs should provide information about the protein’s curvature while upper distance limits (UPL) and dihedral angles (ACO) should retain the native triangular structure of each repeat (scaffold) and the rod-like overall shape. In order to demonstrate that the method is indeed capable of computing the correct structure, we started from a state in which both the PCS and the scaffold restraints are derived from the target structure (input = target structure). In a second set of calculations, we started from a state in which only the PCS restraints are derived from the target while the scaffold restraints are extracted from a (slightly different) model (input ≠ target structure), which corresponds to the more realistic scenario of refining a structure. The advantage of this approach is the generation of easily trackable indicators: the root-mean-square deviation (RMSD) between the resulting structure and the initial model represents the *accuracy* while the RMSD between the 10 best structures ranked by the CYANA target function represents the *precision*, and together they report on the *quality* of the calculation. Using these indicators, coupled to CYANA’s target function, we can identify optimal parameter setups and corresponding limitations.

In what follows, we first describe the parameter optimization for the refinement that was initially used in our computations (input = target structure).

### Optimization of the refinement protocol

We started computations assuming that the backbone is assigned at 100 % for each tagged protein, and optimized four core parameters: (i) the number of molecular dynamics (MD) steps, (ii) the number of computed structures, (iii) the tolerance for PCS data, and (iv) the weights of PCS and UPL restraints.

**Fig. 2:**
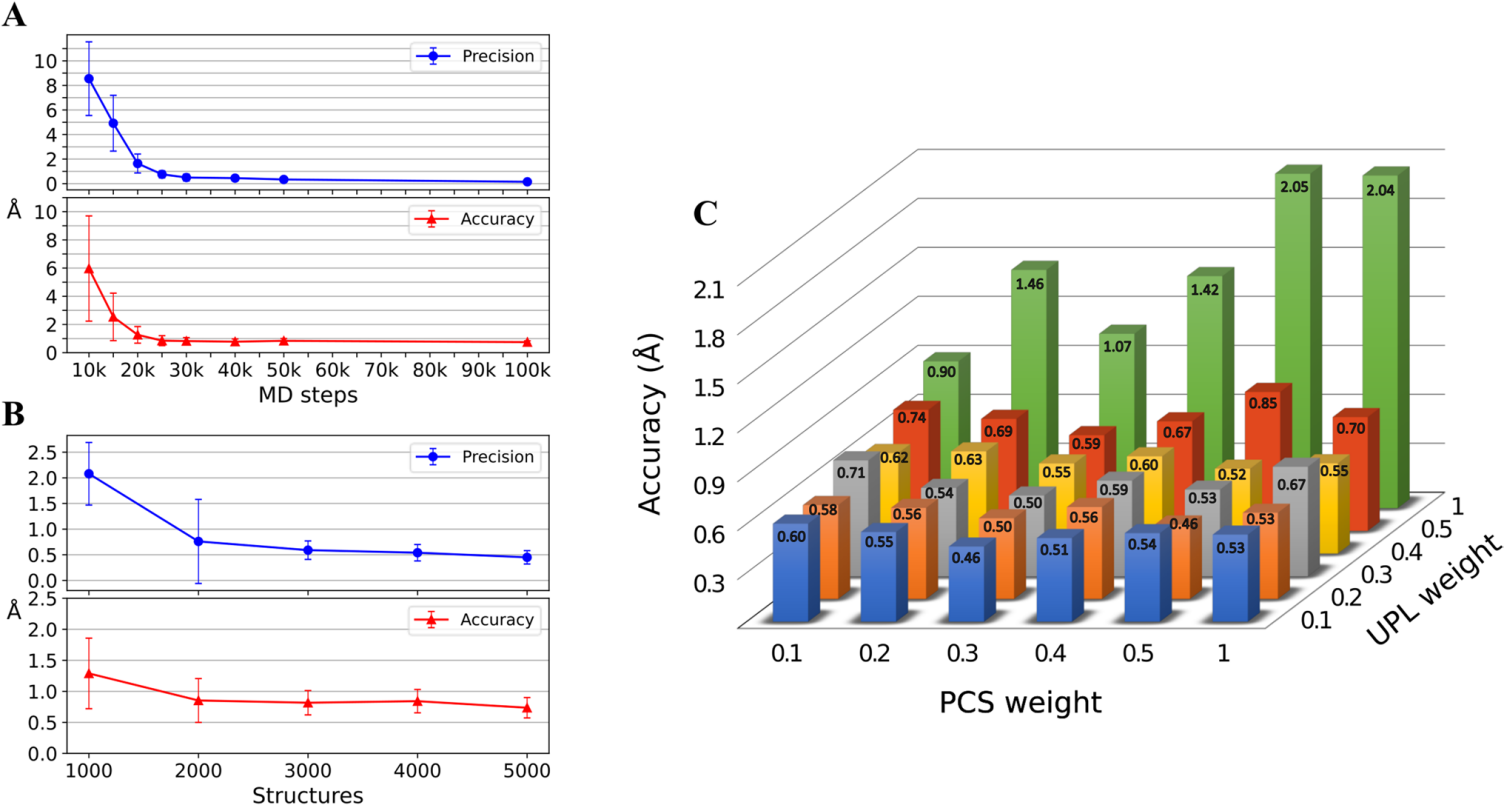
Optimization of core parameters. **a** precision (top) and accuracy (bottom) versus the number of MD steps. Results are shown for calculating 2000 structures with a UPL:PCS weight of 1:0.1. **b** precision (top) and accuracy (bottom) versus the number of calculated structures computed with 25000 MD steps and a UPL:PCS weight of 1:0.1. **c** accuracy versus the UPL and PCS weights when computed with 2000 structures in 25000 MD steps

Increasing the number of time-steps during the MD calculation influences the calculation *quality*. Using the initial default weights of 1.0 for UPL and 0.1 for PCS, we tested values for the number of MD steps in the range between 10000 and 100000. The best quality achieved at reasonable CPU time was observed for 25000 steps, with an accuracy of 0.85 Å and a precision of 0.76 Å (Fig. 2a). In addition, we tested the overall number of computed structures. Since each simulated annealing starts from a (different) random structure, increasing their number potentially allows for sampling a larger conformational space, thus increasing the chances of finding the correct solution. In the range between 1000 and 5000 structures, the best compromise was seen at 3000 structures, with an accuracy of 0.81 Å and a precision of 0.59 Å (Fig. 2b).

Likely the most important parameter is the weight of PCS and UPL restraints, which influences the balance between the scaffold and the experimental PCSs. We varied both weights using values of 0.1, 0.2, 0.3, 0.4, 0.5 and 1.0 and identified the best performance for a weight of 0.1 for the UPL and 0.3 for the PCS restraints, resulting in an accuracy of 0.46 Å and a precision of 0.29 Å (Fig. 2c).

The allowed tolerances for PCSs influence calculations in a complicated manner since the magnitude of the PCS depends on both distance and orientation. Spins remote to the tag in general display smaller PCSs and hence errors in positions may not be corrected if the tolerance is larger than the expected change in PCS for correcting the distance. The three values for tolerances tested (0.02, 0.01 and 0 ppm) showed the expected correlation with accuracy and precision, but since the 0.02 ppm setting already resulted in very good accuracy and precision, 0.45 Å and 0.29 Å, respectively (Fig. S8), and more reasonably reflect experimental uncertainties, we selected 0.02 ppm for the optimized protocol.

These simulations revealed that when utilizing 100 % assignments and using an optimized protocol, structure calculations that are sufficient in accuracy (0.45 ± 0.08 Å) and precision (0.29 ± 0.12 Å), can be performed.

### Analyzing the sampling

The fact that PCSs become fairly ineffective in correcting atom positions for remote atoms suggests that multiple tags are required. Since each new attachment site requires a highly time-consuming process, we set out to theoretically evaluate the optimal tag number and positions.

**Fig. 3:**
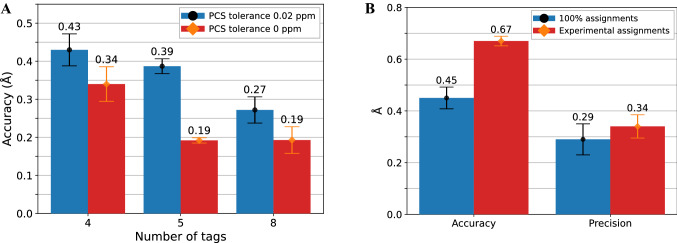
Sampling analysis. **a** accuracy of calculations with additional simulated tags with PCS tolerance set to 0.02 ppm (circles) or 0 ppm (diamonds). **b** accuracy and precision of the positive control with 100 % assigned residues (circles) or with simulated PCS restraints for the previously obtained experimental assignments (diamonds)

To this end we simulated the addition of 1, 2 or 5 additional tags to the three existing tags to result in a total of 4, 5 or 8, respectively, using PCS tolerances of either 0.02 ppm or 0 ppm. The results demonstrate that adding more tags does indeed result in a better convergence but improvements can be marginal (Fig. 3a). While reducing the PCS tolerance has a more substantial effect, imposing zero tolerance is unreasonable since it neglects experimental uncertainties.

Next, we reduced the number of assignments to match our experimental assignments. For each of the experimental YM_4_A attachment sites, we replaced the experimental PCS values with the corresponding virtual ones to closely reflect the experimental conditions. As expected, we observed a decrease in accuracy (0.67 ± 0.04 Å) and precision (0.34 ± 0.09 Å) when compared with the positive control that uses PCS restraints for all residues, but the structure retained sufficient accuracy and precision for the purpose of our study (Fig. 3b).

All parameters affecting the quality of calculated structures were categorized into three classes: class I refers to the *unavoidable* effects and contains the stochastic computational processes (finite number of MD steps and random start structures) and the quality of the input structure; class II refers to the effects due to the experimental *conditions*, such as PCS tolerance and uncertainties in the tag position; class III refers to the effects due to the experimental *data*, such as the number of attachment sites and the number of assigned PCSs.

Our conclusions can be summarized as follows (for more details see the Fig. S9): (a) the increase in accuracy is proportional to the increase in assignments up to 50 % and then flattens out; (b) removing assignments from a specific cap or internal repeat as well as from a specific helix largely does not impact the accuracy; (c) unlike in the *Δχ-*tensor calculation, large PCSs do not provide more information than small ones; (d) tags positioned towards the middle of the protein have a higher impact than those positioned in the caps. This is largely due to the fact that the tensor orientation for that tag is such that it results in a more favorable separation into positive and negative PCSs; (e) an uniform PCS tolerance affects remote spins with small PCSs more than close spins with large PCSs. Therefore, we applied a relative PCS tolerance, in which the tolerance itself is proportional to the magnitude of the corresponding PCS. However, also this approach largely failed to improve the calculation accuracy; (f) a large range of different PCS *weight functions* were tested but offered no improvements.

### Visualizing the trajectory during the simulated annealing

So far, the simulated annealing (SA) procedure remained akin to a “black box” process that revealed little insight into the structural changes during the different phases of the SA and how they are influenced by different choices of parameters. During the cooling phases sidechains need to pack and we suspected that changes in weights for PCS and/or other types of restraints might benefit the process because it could allow the backbone to assume the correct fold while still permitting sidechains to pack and re-adjust to conformations different from the starting model. Hence, we modified the protocol to regularly report coordinates during the SA (see Materials and Methods) allowing us to track structural changes during the different phases to optimize settings for the phases separately.

CYANA’s simulated annealing schedule contains an initial minimization after which the temperature is set to a high value (default 10000 K) followed by a process divided into four phases (Güntert and Buchner 2015): (i) first simulated annealing stage with reduced heavy atom radii; (ii) second simulated annealing stage with normal heavy atom radii and, later, normal hydrogen atom radii; (iii) low temperature phase with increased weight for steric repulsion; (iv) final minimization.

**Fig. 4:**
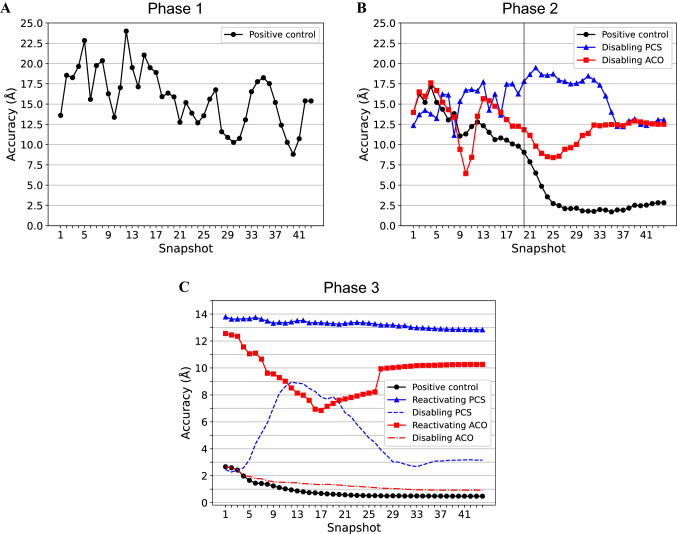
Snapshots from the simulated annealing (SA) trajectory. During the SA a protein snapshot was extracted at a regular number of MD steps and the calculation accuracy was determined by the RMSD between the output and the target structure. The deviation from the target structure is depicted vs. snapshot number (simulation time) during phases 1 (**a**), 2 (**b**) and 3 (**c**) of the CYANA SA run. **a** Phase 1 trajectory of a run including all UPL, ACO and PCS restraints. **b** Phase 2 trajectory from a run that includes all restraints (circles), or runs with all but PCS (triangles) or ACO (squares) restraints. The vertical black line indicates when normal hydrogen radii are activated for calculating Van der Waals repulsions. **C** Phase 3 trajectory using all restraints (black circles), or runs with all but PCS (blue triangles) or ACO (red squares) restraints. Dashed or dashed-dotted lines refer to runs in which PCS or ACO restraints, respectively, were disabled in phase 2 but included in phase 3

We started with a positive control, extracting snapshots every 200 MD steps and plotting the accuracy versus snapshot number (time) (Fig. 4, black line). The results clearly show during phase 1 and the first half of phase 2 a largely unfolded protein (Fig. 4a and b, black line). After restoring normal hydrogen atom radii in the second part of phase 2 (Fig. 4a, black vertical line), the accuracy drastically increases, quickly reaching approx. 2.5 Å. The increased weight for steric repulsion at the low temperature in phase 3 further drives the conformation towards the correct solution with an accuracy of ~ 0.5 Å (Fig. 4c, black line) and no more changes occur during phase 4. A short movie that demonstrates the structural changes during the SA is available in the SI.

In order to investigate the impact of PCSs and dihedral angle restraints (ACO) in phases 2 and 3, we repeated the analysis by disabling one of them in either phase 2 or phase 3 (Fig. 4b and c, blue and red lines). When disabling either of them in phase 2 an overall correct fold is no longer obtained in the second part of phase 2, even when they are reactivated in phase 3, where presumably the low remaining kinetic energy does not allow for larger structural changes. On the other hand, disabling ACOs in phase 3 results in only a moderate decrease in accuracy (~ 1.0 Å), while disabling PCSs results in a considerable loss (~ 3.0 Å). The latter reveals that the system is mainly driven by PCSs, confirming our design.

Several attempts were performed by changing the relative weight of the individual types of restraints in specific phases, without significant improvements (data not shown).

In summary, when testing the procedure by merely recreating the starting structure by using PCSs, we found that some pre-conditions are essential to improve the calculation, namely the number and position of PCS tags and the extent of assignments. Nevertheless, good results were obtained even with a reduced number of tags (3) and partial assignments (33–53 %). Moreover, we identified weights of PCS and UPL restraints as the most influential parameters, and observed that a higher number of starting structures and more MD steps are required for good convergence.

### An iterative procedure for the refinement of PCS-restrained structure calculations

The above-described simulation demonstrated that our protocol was capable of reproducing the correct structure with remarkable accuracy and precision when extracting restraints from the correct structure (input = target). As the second stage, we set up another set of simulations to investigate whether PCSs can drive a system towards the correct solution when the input structure is different from the one that was used to compute the PCS data (input ≠ target). A complication is that the PCS *Δχ-*tensor components depend on the starting and not the target structure, and therefore will be incorrect. To overcome this problem, we introduced the concept of iterative structure calculations: in every cycle, the scaffold restraints (UPL and ACO) are updated based on the newly computed structure and the *Δχ-*tensor is then calculated based on the PCS values from the target structure. A structure calculation is then performed which is used for the next cycle. This allows the incorrect *Δχ*-tensor components and UPL and ACO restraints to be updated steadily while keeping PCS values (that reflect the correct structure) unmodified. The number of PCSs was scaled down to match our experimental assignments.

To this end the reference structure A was manually twisted and dragged to create three different input structures, B, C and D, with a small (0.6 Å), moderate (1.9 Å) and large (2.7 Å) RMSD to structure A, respectively (Fig. S1). Scaffold restraints (UPL and ACO) and *Δχ-*tensor components were extracted from them, following the procedure described above. Finally, all the restraints were used together for the first structure calculation, which was followed by 10 iterative cycles.

**Fig. 5:**
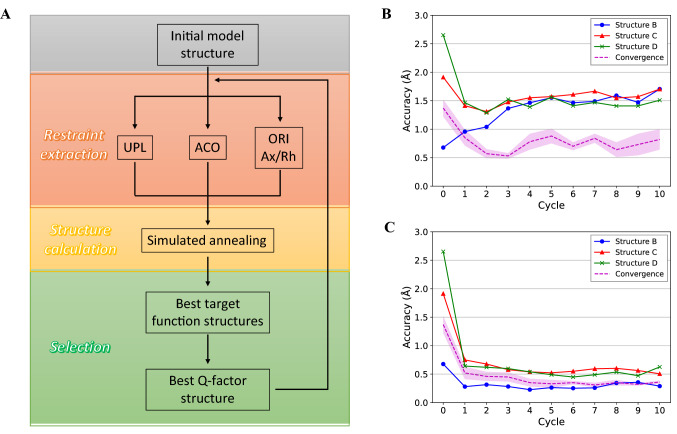
Iterative approach for structure refinement using PCS. **a** Scheme of the protocol used to obtain refined structures in this paper. Panels **b** and **c** illustrate the accuracy of computed structures during the iterative cycles. At the end of every cycle the accuracy is determined relative to the target structure A. Data are shown for each of the three input structures, B, C and D as blue circles, red triangles and green crosses, respectively, while the RMSD between all three output structures is depicted by a dashed line with the shaded region indicating the standard deviation. **b** Accuracy when using similar weights for scaffold and PCS restraints and the conventional protocol for ranking structures. **c** Accuracy when PCSs are strongly favored by an increased weight, scaffold restraints are introduced by regularization, and structures are ranked by their Q-factors

The first set of calculations failed to reproduce the correct structure. Independently from the input structure, each run settled on a ~ 1.5 Å RMSD to the target structure, even when the input itself had a lower initial RMSD (Fig. 5b). This suggested that the relative weight of the restraints to maintain the overall geometry of the modules was too high compared to those representing the PCSs. Thus, we alternatively restrained each module using CYANA’s *regularize* macro (Gottstein et al. 2012): therein, each atom is allowed to move during the calculation at most by a user-defined amount (default: 0.3 Å) from its initial position in the starting structure. This efficiently maintains the structure of each module close to its input conformation while allowing small adjustments to take place, and is more flexible than a complete freezing of the module structures. To further improve the impact of PCSs, we increased the PCS and UPL weights to 30 and 1 (from 0.3 and 0.1), respectively (Fig. S10). Finally, we ranked computed structures not only by CYANA’s target function, which takes many different interactions into account, but based on Q-factors, as defined by

$$Q=\sqrt{\frac{{\sum }_{i}\left[{\left({\sum }_{m}\left[PC{S}_{m,i}^{exp}-PC{S}_{m,i}^{calc}\right]\right)}^{2}\right]}{{\sum }_{i}\left[{\left({\sum }_{m}\left[PC{S}_{m,i}^{exp}\right]\right)}^{2}\right]}}$$

where the indices *i* and *m* run over atoms and models respectively. Q-factors represent the quality of the *Δχ-*tensor fit and hence how PCSs computed from the obtained structures agree with the input PCSs. Using this optimized protocol (Fig. 5a), all three input structures B, C and D converged towards A, settling on the previously observed accuracy threshold of ~ 0.5 Å (Fig. 5c). We like to emphasize here that scaffold ACO restraints were applied across the *entire* sequence (and not just to portions of the structure), because the PCSs in the end will correct for ACO restraints from an incorrect model once they are properly weighted by taking the relative number of scaffold and PCS restraints into account.

We then thoroughly tested the impact of different input structures on the calculation quality. Details of the analysis are described in the Supplementary Information (Fig. S11). To summarize: (a) the calculation quality depends on the RMSD between starting and target structure. But even when using starting models that differ significantly from the target, the accuracy increases with every cycle, indicating a correct solution would simply require more cycles; (b) the computation is capable to “fix” a local structure distortion in the starting model (introduced via changes to upper-distance restraints), such as a partially unpacked cap or a bent helix; (c) sizable deviations in the dihedral angles from the true structure (introduced via changes to dihedral-angle restraints) are still corrected during the computation.

Due to their repetitive sequence, repeat proteins are difficult to assign. Accordingly, we tested the impact of wrong assignments by swapping assignments between one or more fragments. We observed that the calculation fails to reach the correct solution. Interestingly, it is possible to spot such errors by comparing Q-factors associated with the swapped assignments (data not shown).

To conclude, when investigating how PCSs can help in refining structures, we identified the correct balance between scaffold and PCS restraints as the most dominant factor. The regularize approach is particularly influenced by the PCS and UPL weights with good settings of UPL weights of 1 and PCS weights of 30–50, depending on the PCS quality. Despite the high weight of PCS restraints, major changes required in the restrained scaffold would result in a high penalty that could prevent the calculation to reach the correct solution. Therefore, we designed an iterative process that selects structures for best fit of PCS restraints while still allowing for small adjustments of the scaffold in each cycle.

### Determination of the structure of YM_4_A using the optimized protocol

**Fig. 6:**
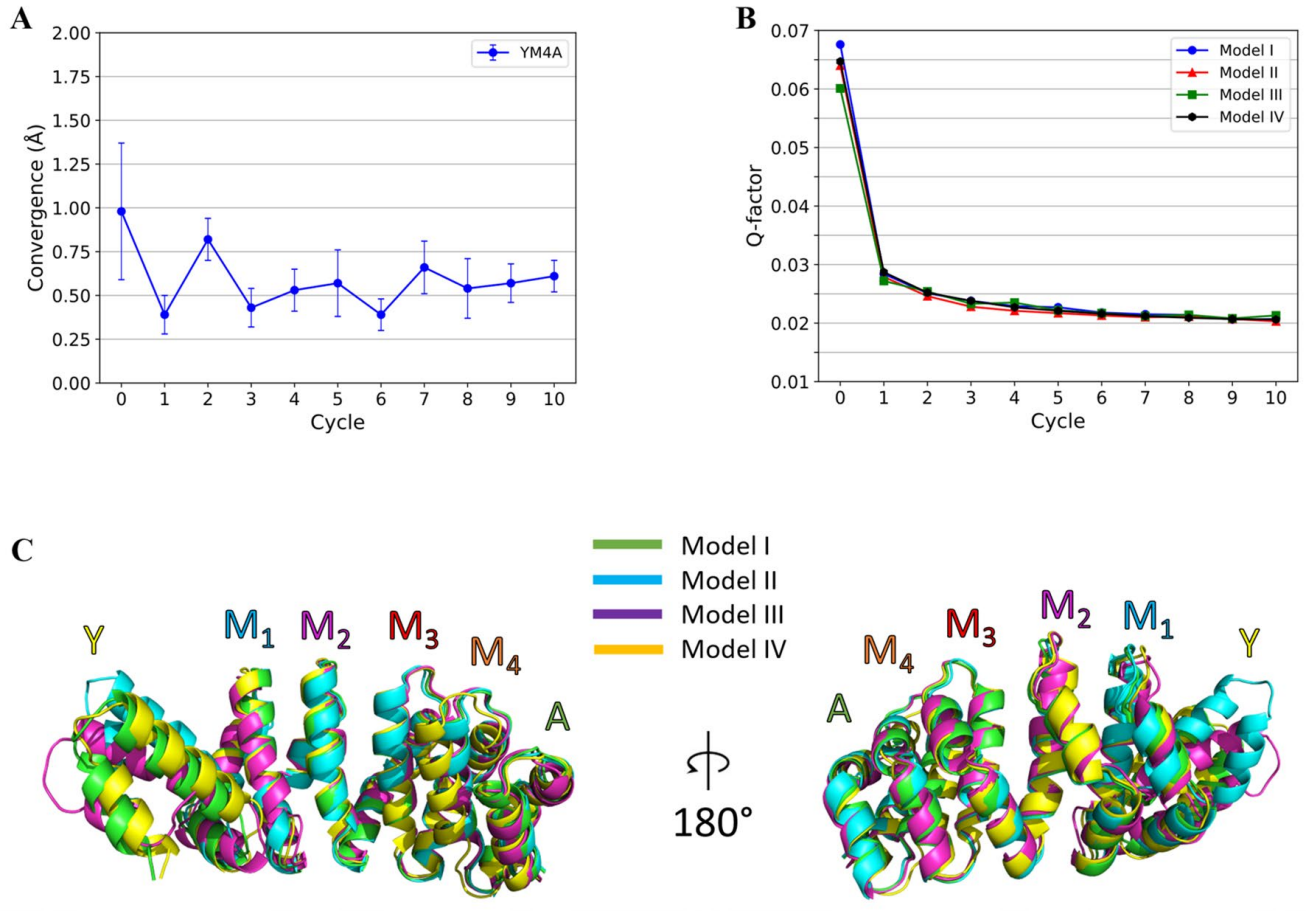
Details from the PCS-refinement of YM_4_A. **a** Convergence of computed structures as represented by the RMSD between structures calculated starting from the four models, after every cycle, but not including the Y cap and the first internal module. Error bars indicate the standard deviations. **b** Q-factors for PCS data derived from YM_4_A spin-labeled in the A-cap via S21C, as calculated with Paramagpy. Data are shown for calculations starting from the four models. **c** Cartoon presentations of structures after the 10th cycle for each of the four calculations with a different input model structure. Atom positions are aligned across the entire sequence

As the third and last stage, we used the iterative procedure to calculate for the first time the solution structure of a dArmRP, YM_4_A. Using experimentally determined PCS restraints (Fig. S5, Tables S13 and S14), we observed convergence of the calculations when starting from four different input models derived from known crystal structures of homologous dArmRPs (see Materials and Methods). After 10 iterations each, all the final structures displayed the typical dArmRPs fold while moderately varying in the protein supercoil when compared with their homologous crystal structures. When comparing superpositions of the entire sequence and of the sequence excluding the Y-cap and the first module we noticed significantly reduced values for the RMSD in the latter, which is related to the fact that the Y cap does not pack stably against the remainder of the protein. For this reason, the Y cap and the first module were removed from the subsequent analysis (Fig. 6c). To account for this problem we have developed in the meantime a modified version of the cap devoid of this issue (manuscript in preparation). Convergence between the four different inputs improved during the cycles (Fig. 6a). Q-factors also steadily decrease throughout the cycles, indicating that structures from each new cycle fit better with the experimental data (Fig. 6b and Fig. S12). Finally, we further tested the reproducibility by repeating the four cyclic calculations using two different random seeds that are used by CYANA to randomly create the starting coordinates. The RMSD between the computed structures after 10 cycles from the same input data but starting with different seeds reveals good convergence with an overall RMSD of 0.52 ± 0.12 Å (Table S15).

#### Discussion & conclusions

PCSs play a well-established role in protein structure calculations, where they are usually used to augment existing structural information confirming known structures, performing refinements, and orienting domains relative to each other. In this work we emphasize the additional possibility of determining protein structures from models in the complete absence of sidechain assignments - a situation in which too few of the structurally important long-range NOE restraints are available for classical NOE-based protocols.

Unfortunately, computing structures from PCS restraints is complicated by the fact that, even when the diamagnetic state is fully assigned, transfer of assignments to the paramagnetic state is often not trivial. Herein, the assignment of paramagnetic spectra follows an iterative procedure, in which an initial set of unambiguous assignments, concluded either from the slope of lines connecting peaks from the diamagnetic and the paramagnetic states, or from peaks in isolated regions, is used to approximate the *Δχ-*tensor, which in turn is used to obtain even more assignments. To increase the number of initial unambiguous assignments, single amino acid labelling is employed. The repetitive nature of repeat proteins without knowledge of the precise structure unfortunately hampered this process in our case. We therefore exploited the modular nature of dArmRPs by expressing a series of smaller constructs with conserved attachment sites of the paramagnetic moieties to guide the assignment procedure. However, even with this approach, only a limited set of assigned PCSs was obtained.

Existing protocols for PCS-based structure calculations employ a large number of PCS restraints and hence a sizeable number of assignments to reduce *Δχ-*tensors tensor ambiguities. However, this is possible only for well-behaving proteins while for larger or more complex systems PCSs need to be complemented with NOEs-derived distance and dihedral angles restraints (Banci et al. 2002), or RDCs and PREs. As an alternative, robust ROSETTA-based protocols have been developed to support the fragment picking procedure with PCS and CS data, that require only sparse PCSs (Kuenze et al. 2019; Pilla et al. 2016). Although in general these approaches work very well, they fail to achieve the high accuracy desired in this work. In contrast, the protocol presented in this paper utilizes PCSs as the only source of experimental restraints, exploiting known structural features of a starting homology model to frame a scaffold that is subsequently iteratively refined. Moreover, the procedure requires only a limited set of assigned PCSs, in our case ranging from 33 to 53 %, thus overcoming assignment issues in crowded spectra, but still achieves high accuracy and precision. Importantly, the impact of additional data, which often can only be obtained by cumbersome additional biochemical or spectroscopic work, can be easily assessed by performing computations with an appropriately scaled amount of PCS data. This should help in deciding whether time-consuming additional assignments can be expected to provide significant improvements.

We spent considerable effort into modifying existing protocols, much of which did not result in significant improvements. The breakthrough in correctly harnessing PCSs was acquired realizing that for successful refinement a balance between the predetermined scaffold and the experimental PCS restraints must be maintained. An over-restrained scaffold prevents PCSs from really driving the refinement (accuracy loss), while an excessively loose scaffold ensues multiple solutions with similar energies (precision loss). CYANA’s regularize approach together with appropriate PCS and UPL weights are key to good performance. Even when using proper relative weights of scaffold and PCS restraints, performing the refinement in a single step would limit the extent by which the scaffold can be rearranged because violation of scaffold restraints would at some point outweigh the impact from PCS restraints. Therefore, we developed the procedure into an iterative process that allows stepwise gradual adjustments of the scaffold which in their sum still can present significant changes in the scaffold, and designed a selection based on Q-factors ensuring that the final structure presents the best fit to the PCS.

The protocol presented in this paper was initially developed to tackle specific issues concerning the tertiary structure of repeat proteins. However, we quickly realized that the iterative methodology can also be extended to proteins with different folds. We suspect that in order to refine proteins of different structures, parameters in the protocol have to be fine-tuned to ensure robust and reliable refinement with the available experimental data. However, thanks to extensive automation of procedures by the scripts developed in this work (see SI), the user only needs to provide the sequence, a set of PCSs and a starting structure to initialize the generation of a positive control. Virtual PCSs can be easily computed from a model (Materials and Methods), which represents the target structure in this case, and the same structure can then be modified, representing the input. Fine-tuning can then be achieved by performing automated calculations in which the core parameters discussed in this paper are varied and their impact on the accuracy is accessed. Parameters with highest impact are the PCS and UPL weights, the regularize value that defines how much each atom may deviate from the input structure, the uncertainty in the position of the paramagnetic metal and the number of iterations (the full list is presented in the protocol capture in the SI). We like to reemphasize that the positive control will reveal whether refinement with the finally chosen parameter will result in correct structures.

For proteins without intrinsic metal binding capabilities suitable lanthanide-chelating tag attachment sites are often found in time-consuming trial-and-error searches. Any method providing good predictions of suitable attachment sites would be of great value, and therefore testing how well a given attachment site would reproduce the input structure is very helpful in determining the information content of the PCSs generated by such a site. In the case of tags with a single attachment site, such as the Tm-3R4S-DOTA-M7-thiazole used in this work, unstable association with the protein surface may occur, therefore triggering motional averaging and hence very small PCS. While the protocol does not help in predicting whether a tagging site will be well-behaved in that sense, it does provide insight into whether PCS due to a specific tagging site could add *meaningful* data, as opposed to just add *more* data. With the described protocol the user can easily select multiple different attachment site candidates in various combinations, compute the resulting PCSs based on an existing *Δχ-*tensor template, and assess how they influence the structure calculation. This should help guiding the initial tagging scheme. To reiterate, the procedure is highly automated, and hence new tagging sites can be easily screened.

The most widespread method to calculate solution NMR structures is through NOEs. This strategy, however, requires a considerable number of assignments of both backbone and sidechains resonances. Unfortunately, for many proteins, in particular those large on the NMR scale or those that display line-broadening due to aggregation or structural instability, nearly complete assignments are difficult or even impossible. A valuable alternative to PCSs are residual dipolar couplings (RDCs) (Bax et al. 2001; Prestegard et al. 2004; Tjandra and Bax 1997). Similarly to PCS they allow to refine atom positions against a common reference frame, thereby providing long-range information. In our experience, however, experiments for measuring RDCs are far more difficult to perform on large proteins, because the rather long pulse sequences are prone to relaxation. In addition, data are difficult to evaluate in crowded spectra, as is the case for repeat proteins, where the PCS tags result in much improved signal dispersion albeit at the expense for additional (often challenging) assignments.

Often, crystal structures of homologous proteins are available that may serve as templates for PCS-based refinements. The recent progress in artificial-intelligence-supported structure prediction, as demonstrated by the AlphaFold team (Senior et al. 2020), indicates that predictions suitable as template structures for PCS-based refinements may become available in the future, leaving us with the task to refine or to validate them. Structure refinement as described in this work allows to exploit the long-range nature of PCS to obtain accurate backbone structures without any sidechain assignment. Backbone assignments are much easier to obtain for large proteins than the corresponding sidechains assignments, in particular when considering the need for perdeuteration. In our test studies, we employed only amide backbone PCSs and obtained remarkable accuracy and precision, although an only approximately correct starting model structure was available. Such backbone structures can then serve as scaffolds for sidechain modelling through comparative modelling (Bender et al. 2016).

In our project, crystal structures of homologous dArmRPs proteins were available. The combination of crystallographic data that are used to restrain the individual modules, and PCS-based NMR refinement provides an avenue to determine structures of these proteins in solution, devoid of packing artifacts. This task, while not entirely impossible, would have been much more challenging without the structural insight from crystallography. In addition, this PCS-based protocol can be used to verify or even correct structures derived by methods that are potentially prone to artifacts due to crystal packing in X-ray crystallography or specimen preparation for cryo-EM (Elmlund et al. 2017).

## Supplementary Information

Below is the link to the electronic supplementary material.

Supplementary file 1 (DOCX 52821 KB)

Supplementary file 2(XLS 12 KB)

Supplementary file 3 (xlsx 10 KB)

Supplementary video (mp4 372787 KB)

## Data Availability

The Supporting Information is available free of charge on the Publications website. ^1^H and ^15^N chemical shifts and their corresponding pseudocontact shifts of YMA- and YM_4_A-type mutants have been deposited in the BMRB data base under accession codes 50824, 50825, 50826, 50827, 50828 and 50829.

## Code availability

A tutorial containing a protocol capture with the relevant Python scripts and Cyana macros is available on Github (https://github.com/Evets90/Iterative_PCS_refinement). Further Python scripts and CYANA macros are available upon request.
