# Supplementary material for: An automated iterative approach for protein structure refinement using pseudocontact shifts
Source: J Biomol NMR. 2021 Aug 2;75(8-9):319–34. doi: 10.1007/s10858-021-00376-8 (PMC8473369; doi:10.1007/s10858-021-00376-8)
Supplement: Supplementary file 1 — (DOCX 52821 KB) [file 10858_2021_376_MOESM1_ESM.docx]

**Supplementary information**

**An automated iterative approach for protein structure refinement using pseudocontact shifts**

Stefano Curcuzza^1^, Peter Güntert^2^, Andreas Plückthun^3^, Oliver Zerbe^1^*

^1^ Department of Chemistry, University of Zürich, Winterthurerstrasse 190, 8057 Zürich, Switzerland

^2^ Institute of Biophysical Chemistry and Center for Biomolecular Magnetic Resonance, Goethe University Frankfurt, Max-von-Laue-Straße 9, 60438, Frankfurt am Main, Germany

Laboratory of Physical Chemistry, ETH Zürich, Vladimir-Prelog-Weg 2, 8093 Zürich, Switzerland

Department of Chemistry, Tokyo Metropolitan University, 1-1 Minami-Osawa, Hachioji, Tokyo 192-0397, Japan

^3^ Department of Biochemistry, University of Zürich, Winterthurerstrasse 190, 8057 Zürich, Switzerland

*corresponding author:

Email addresses of corresponding authors: [oliver.zerbe@chem.uzh.ch](mailto:oliver.zerbe@chem.uzh.ch)

This file contains:

- Supplementary Figures S1, S2, S3, S4, S5, S6, S7, S8, S9, S10, S11, S12 and S17
- Supplementary Tables S13, S14 and S15
- Caption of Supplementary Video S16


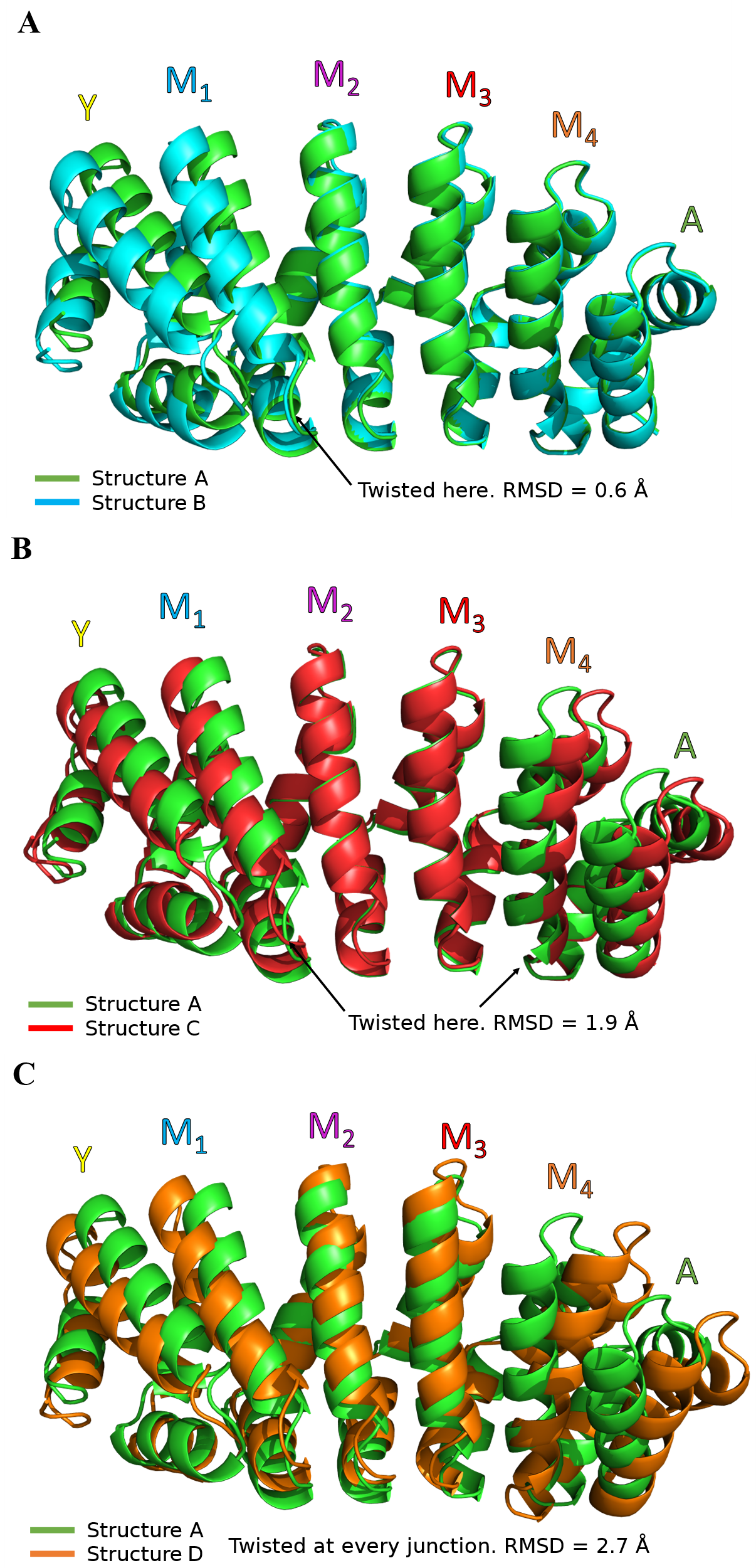


**Fig S1** Cartoon representation of the four model structures of YM_4_A. Structure A (green) was twisted and dragged in three different ways to produce three additional model structures, referred to as structure B, C and D. **a** alignment of structure A (green) and structure B (cyan). The junction between twisted modules is indicated with an arrow. **b** alignment of structure A (green) and structure C (red). **c** alignment of structure A (green) and structure D (orange)


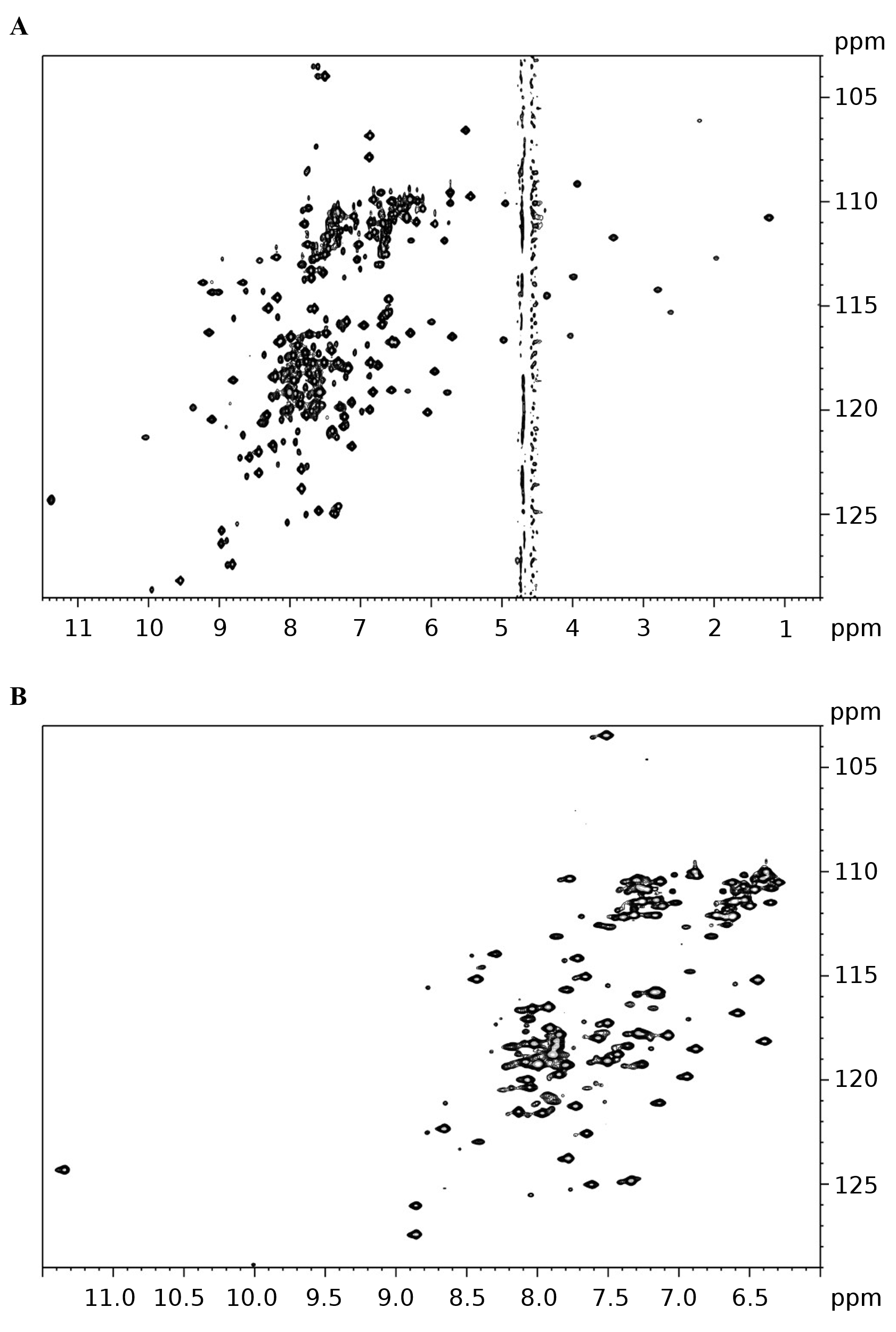


**Fig S2** Examples for unsuitable attachment sites. [^15^N,^1^H]-HSQC spectra of YMA tagged with Tm-4R4S-DOTA-M8 displaying spectra of poor quality. **a** spectrum of (M)Q5C, showing multiple sets of peaks, indicating two different modes by which the tag associates with the protein surface. **b** spectrum of (M)D9C exhibiting very small PCS shifts due to motional averaging.


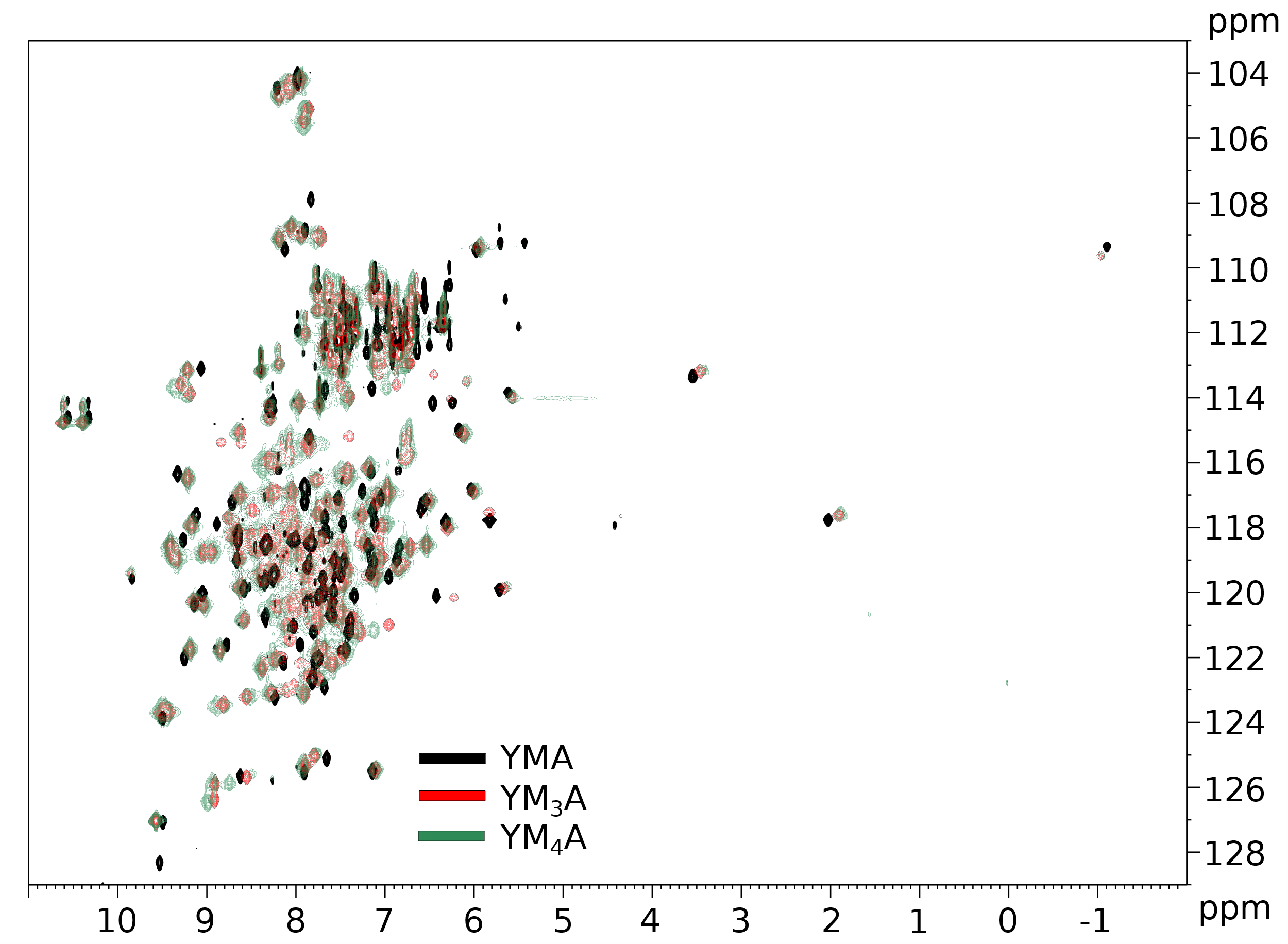


**Fig S3** Superposition of [^15^N,^1^H]-HSQC spectra of (A)S21C YM_n_A tagged with Tm-4R4S-DOTA-M8, where n=1,3,4 in black, red and green, respectively.


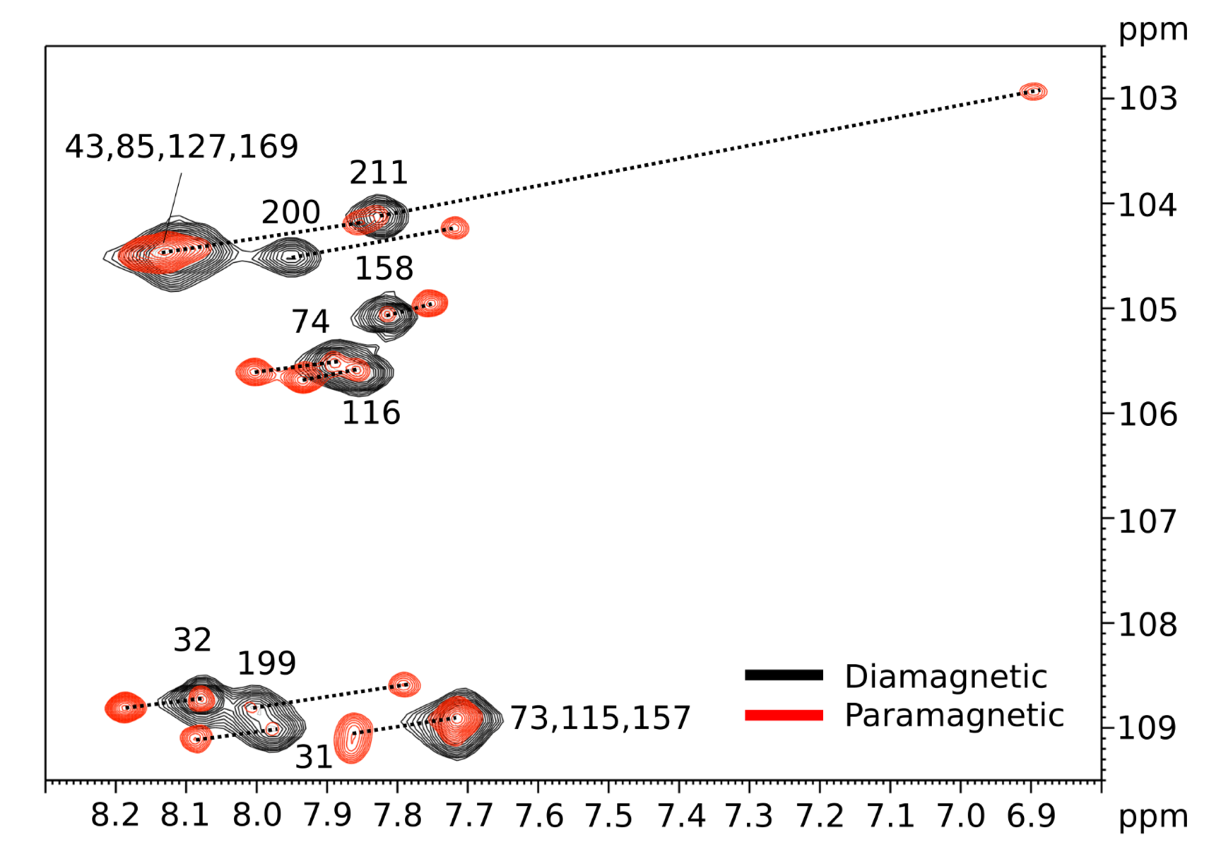


**Fig S4** Initial assignments in isolated regions. Glycine region of [^15^N,^1^H]-HSQC spectra of (M3)S21C YM_4_A tagged with Lu-3R4S-DOTA-M7Thiazole (black) or with Tm-3R4S-DOTA-M7Thiazole (red). Diamagnetic assignments are annotated on top of their respective signals while induced PCSs are depicted by a dotted line. Assignments of multiple single residues to the same position are separated by a comma.

**
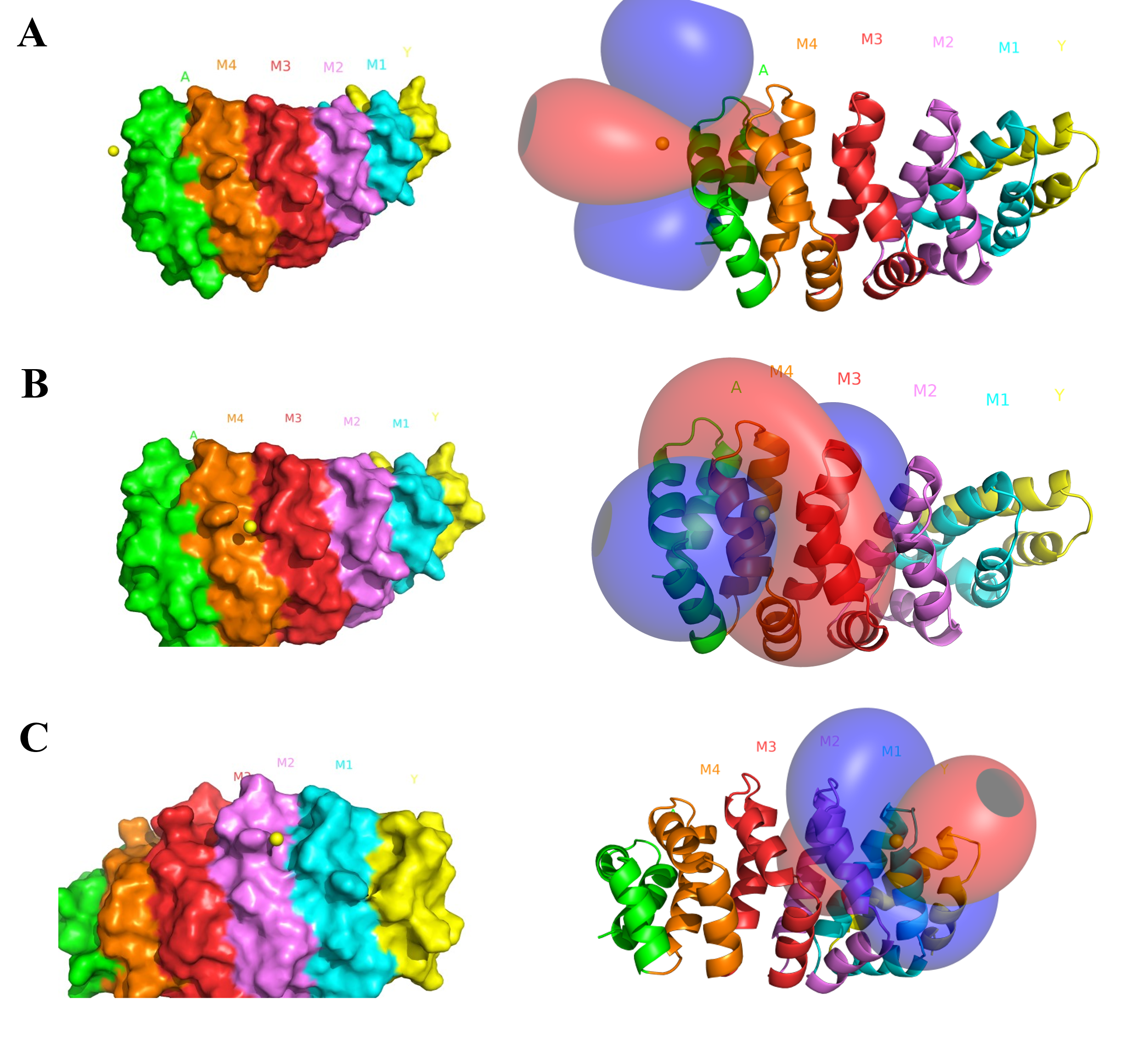
**

**Fig S5** YM_4_A models coupled to the paramagnetic tag at different attachment sites. Structure A (see Materials and Methods) was used as input structure to calculate the *Δχ-*tensor components using experimentally determined PCS values. The thulium atom in the paramagnetic tag is represented as a yellow sphere while each YM_4_A module and the caps are color-coded differently. On the left, the metal center position is depicted relative to the surface. On the right, the *Δχ-*tensor is shown as an isosurface, with positive lobes depicted in blue and negative lobes depicted in red. **a** first attachment site, (A)S21C. **b** second attachment site, (M3)S21C. **c** third attachment site, (M1)Q18C


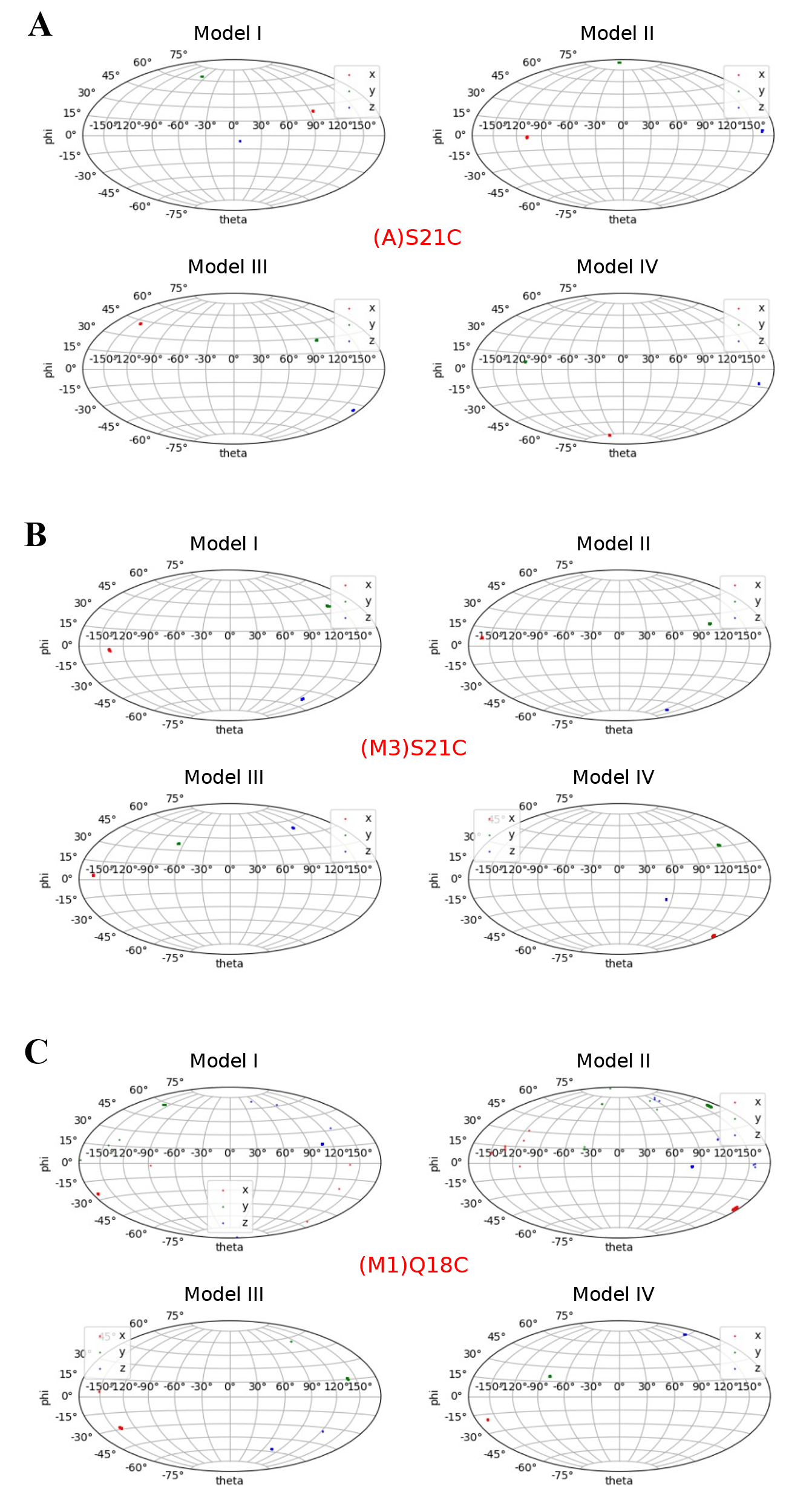


**Fig S6** Quality of YM_4_A *Δχ-*tensors fits estimated from a Monte-Carlo analysis performed with Paramagpy. The analysis was performed using single fit mode through 200 iterations with an experimental uncertainty (σ) of 0.005 ppm and 100% sample fraction, with the grid search centered on the x, y, z coordinates of the Cα atom of the attachment site. For each iteration, the resulting *Δχ-*tensor’s principal axis components are represented as a single dot in a Sanson-Flamsteed equivalent projection for the x (red), y (green) and z axis (blue) components. In this type of projection, the axes of the tensor penetrate the surface of a unitary sphere, and the penetration points can be identified on the projection, thus enabling to asses if multiple fit with added noise results in robust results (dots close by) or poor fits (dots spread). For each attachment site, the fit was tested on the four available model structures I, II, III and IV (see Materials and Methods), located in the top-left, top-right, bottom-left and bottom-right corners, respectively. **a** first attachment site, (A)S21C. **b** second attachment site, (M3)S21C. **c** third attachment site (M1)Q18C


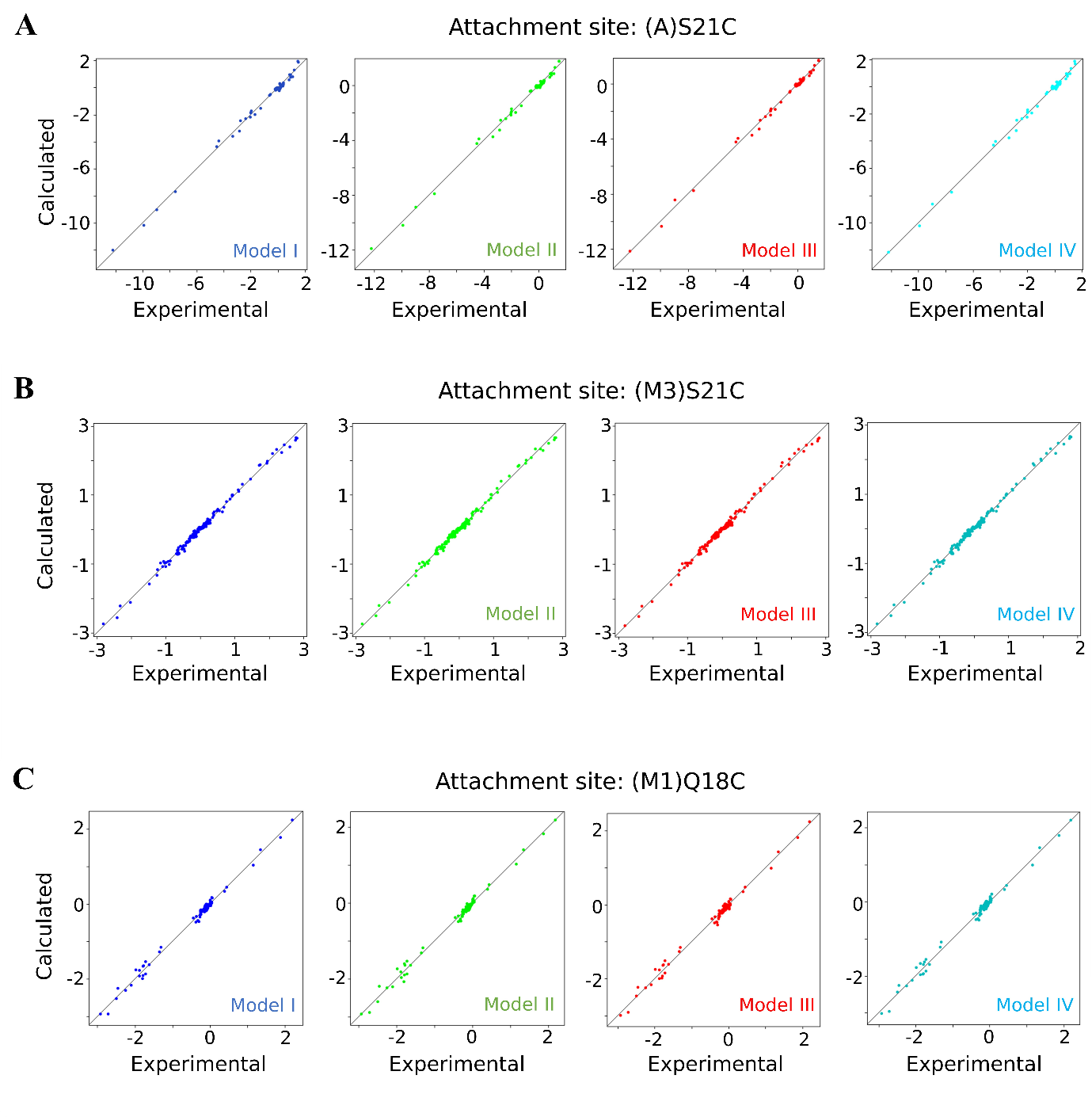


**Fig S7** Correlations between experimental and theoretical PCS back-calculated with Paramagpy from the initial four model structures (I-IV) used in the YM_4_A structure refinements for PCS data obtained from three differently tagged proteins **a** first attachment site, (A)S21C. **b** second attachment site, (M3)S21C. **c** third attachment site, (M1)Q18C.


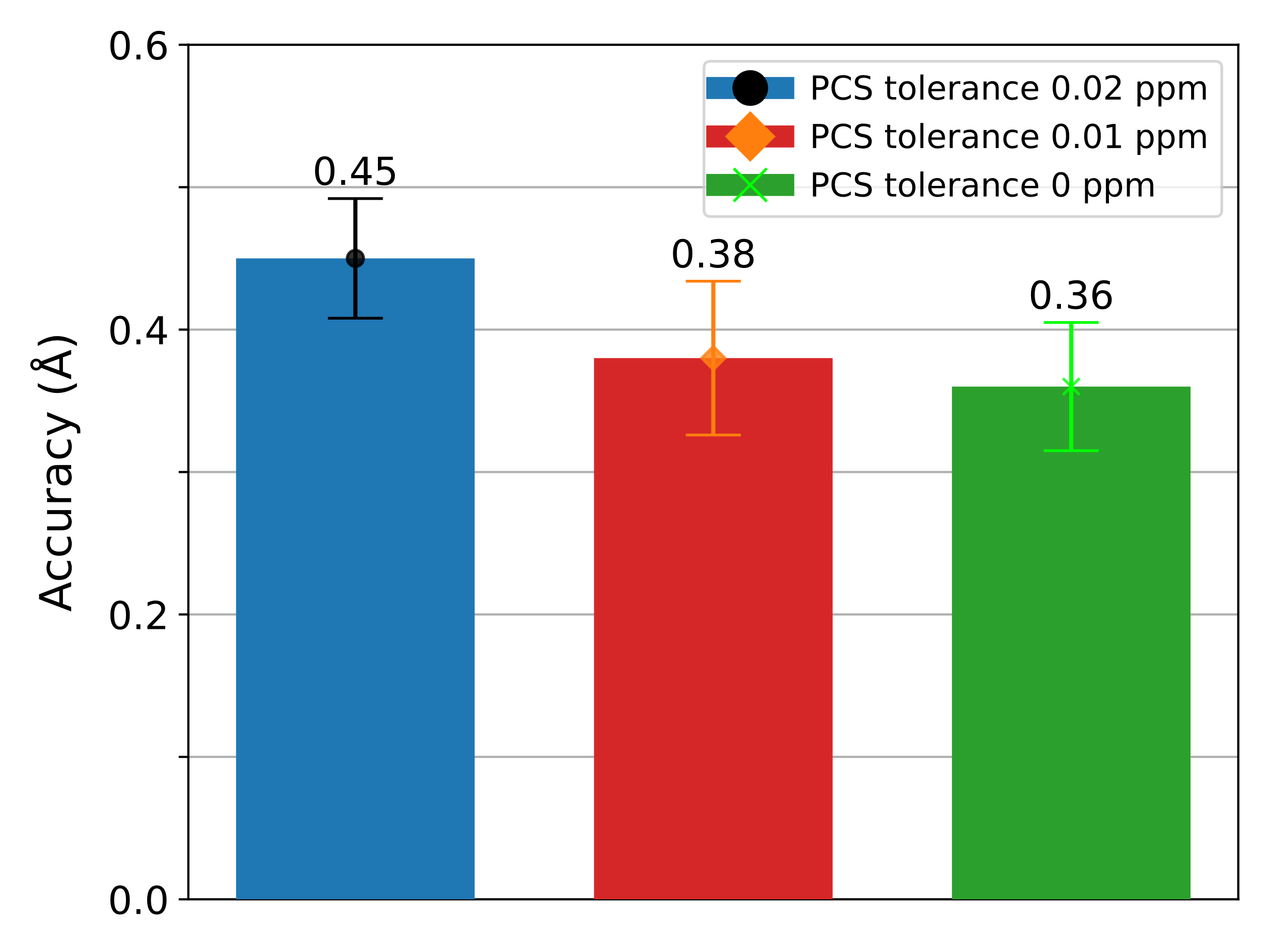


**Fig S8** Testing accuracy as a function of the PCS tolerance in the non-iterative procedure. Each calculation was performed by annealing 3000 random structures in 25000 MD steps with PCS and UPL weights of 0.3 and 0.1, respectively, employing 100% assignments, setting the PCS tolerance to 0.02 ppm (circle), 0.01 ppm (diamond) or 0 ppm (cross).


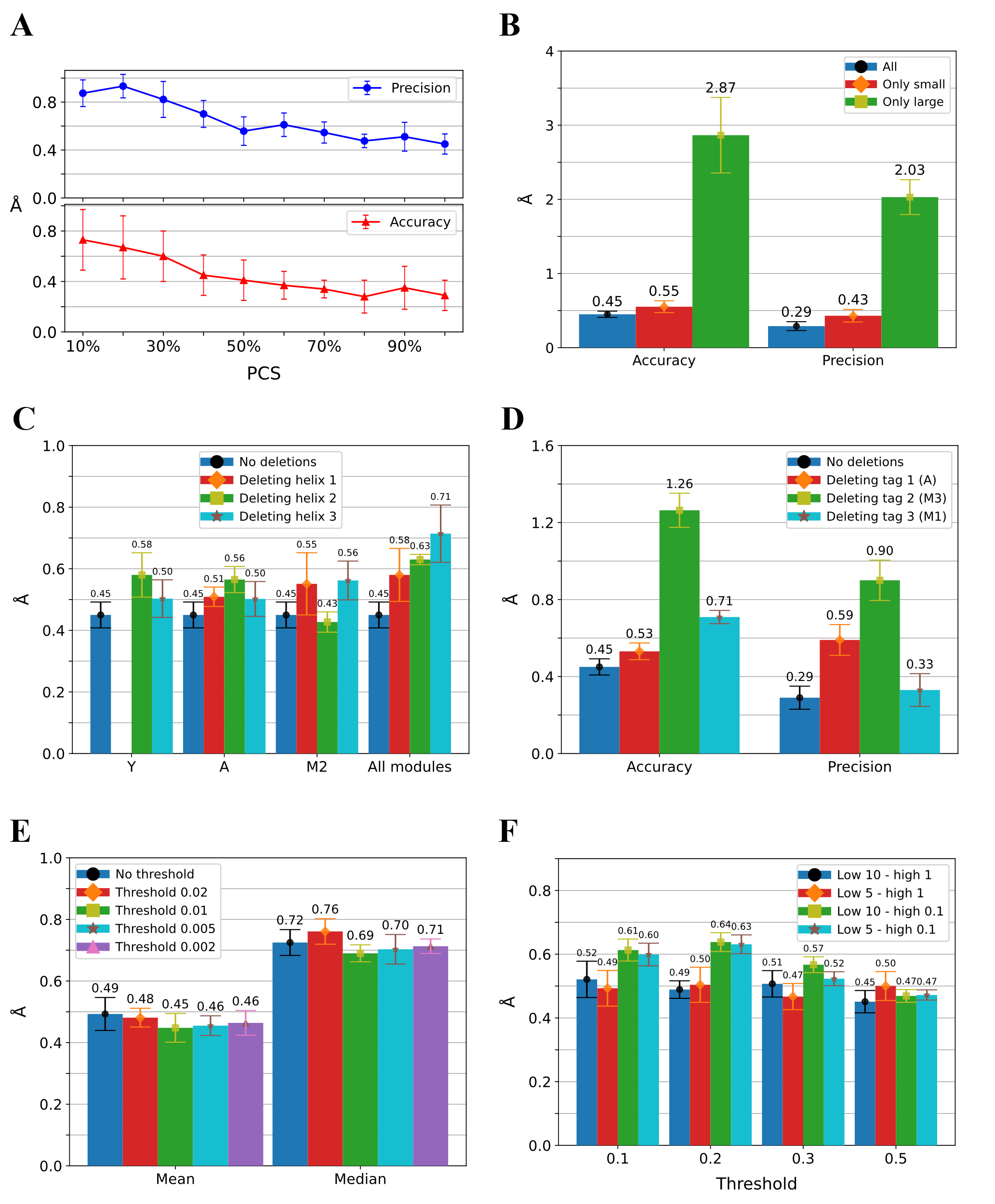


**Fig S9** Details of simulations in the context of the non-iterative approach. Unless specified, all the calculations were performed as described in Materials and Methods. **a** simulating the impact of incomplete PCS data. A random amount of PCS restraints between 10% and 90% in 10% steps were deleted in the data from the three attachment sites and plotted against the resulting accuracy (triangles) and precision (squares). **b** simulating the impact of small (< 1 ppm, diamonds) versus large (≥ 1 ppm, squares) PCSs. **c** simulating the impact of deleting all PCS data from specific helices. The positive control with no deletions (squares) was matched with calculations in which helix 1 (diamonds), 2 (squares) or 3 (stars) were deleted in the Y cap, A cap, internal module M_2_ or in all modules, from left to right, and plotted against the accuracy. Note that the Y cap does not possess a helix 1. **d** simulating the impact of individual tags. The positive control with no deletions (squares) was matched with calculations in which tags in the first (diamonds), (A)S21C, second (squares), (M3)S21C, or third (stars), (M1)Q18C, attachment sites were deleted and plotted against the accuracy (left) and precision (right). **e** simulating the impact of relative PCS tolerances on the accuracy. The standard PCS tolerance of 0.02 ppm was compared to the mean (left) or median (right) PCS value among all attachment sites to calculate a relative PCS tolerance that was then applied to all other PCS values. Thereby, large PCSs will have a large tolerance while small PCSs will have a small tolerance. In order to remove unrealistically small tolerances, all calculated tolerances smaller than the threshold of 0.02 (diamonds), 0.01 (squares), 0.005 (stars) or 0.002 (triangles) ppm were set to the threshold itself. **f** simulating the impact of different weighting function on accuracy. In order to give more impact to small PCS, an inverse weight function was applied to all PCS values below a threshold of 0.1, 0.2, 0.3 or 0.5 ppm, from left to right. These were assigned a weight referring to the class “small PCS” and to the remaining values the weight referring to the class of “large PCS”. Four combinations were tested: “small PCS” 10 – “large PCS” 1 (circles), “small PCS” 5 – “large PCS” 1 (diamonds), “small PCS” 10 – “large PCS” 0.1 (squares) and “small PCS” 5 – “large PCS” 0.1 (stars).


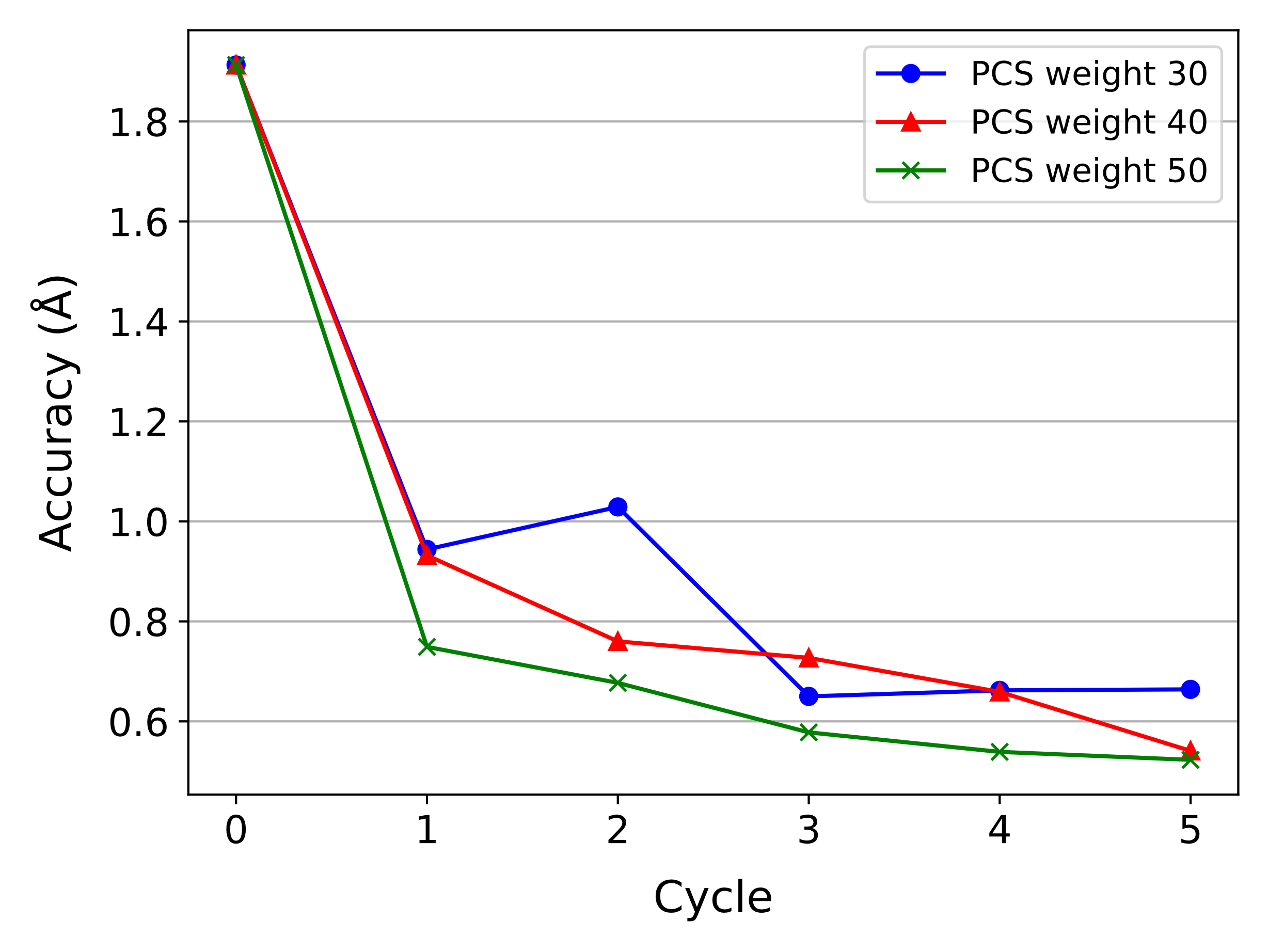


**Fig S10** Testing the accuracy as a function of PCS weight in the iterative procedure. Each calculation was performed using C as the input and A as target structures, annealing 500 structures in 25000 MD steps for each of the 5 iterative cycles, and setting the PCS weight to 30 (circles), 40 (triangles) or 50 (crosses). Scaffold restrains were implemented using CYANA’s *regularize* macro, and structures were ranked based on their average Q-factors.


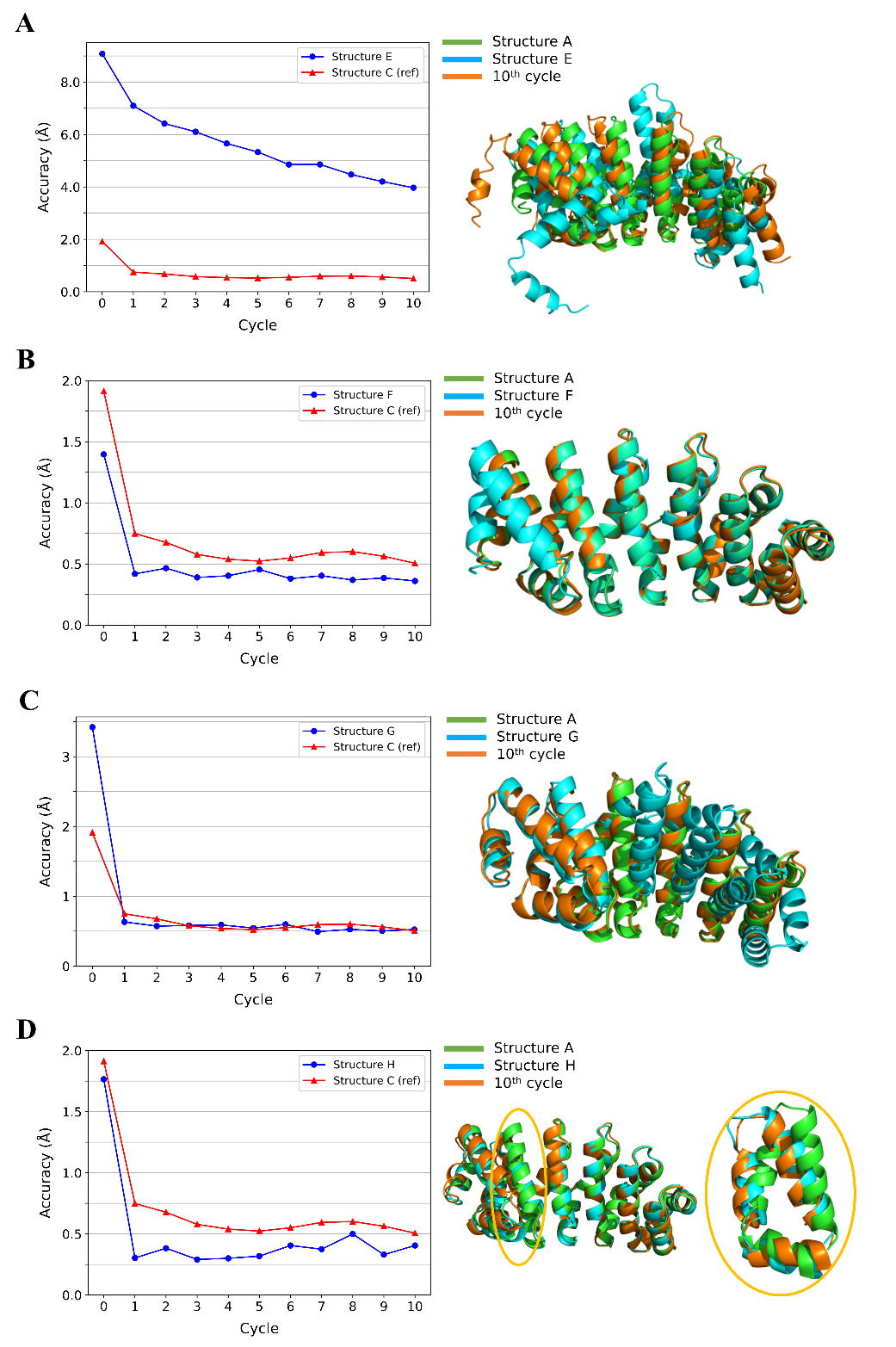


**Fig S11** Testing the performance of the iterative procedure by evaluating the accuracy with different input structures. All calculations were performed annealing 500 structures in 25000 MD steps for 10 cycles. The reference calculation (triangles) is performed with structure C as input and structure A as target (medium difference) and is shown in all graphs for comparison. The cartoon on the right is a superposition of the input, target and final structure after 10 cycles of refinement. The starting structure E was designed to uniformly display large differences for all modules. Structure F contains a (partially) unpacked Y cap. Structure G was created to contain a big change in the protein curvature at the M_1_-M_2_ junction. Structure H contains a bent helix in H3 of module 1. **a, b, c** calculations using structure E , F and G as inputs (circles), respectively and structure A as the target. **d** calculations using structure A as input (circles) and structure H as target.


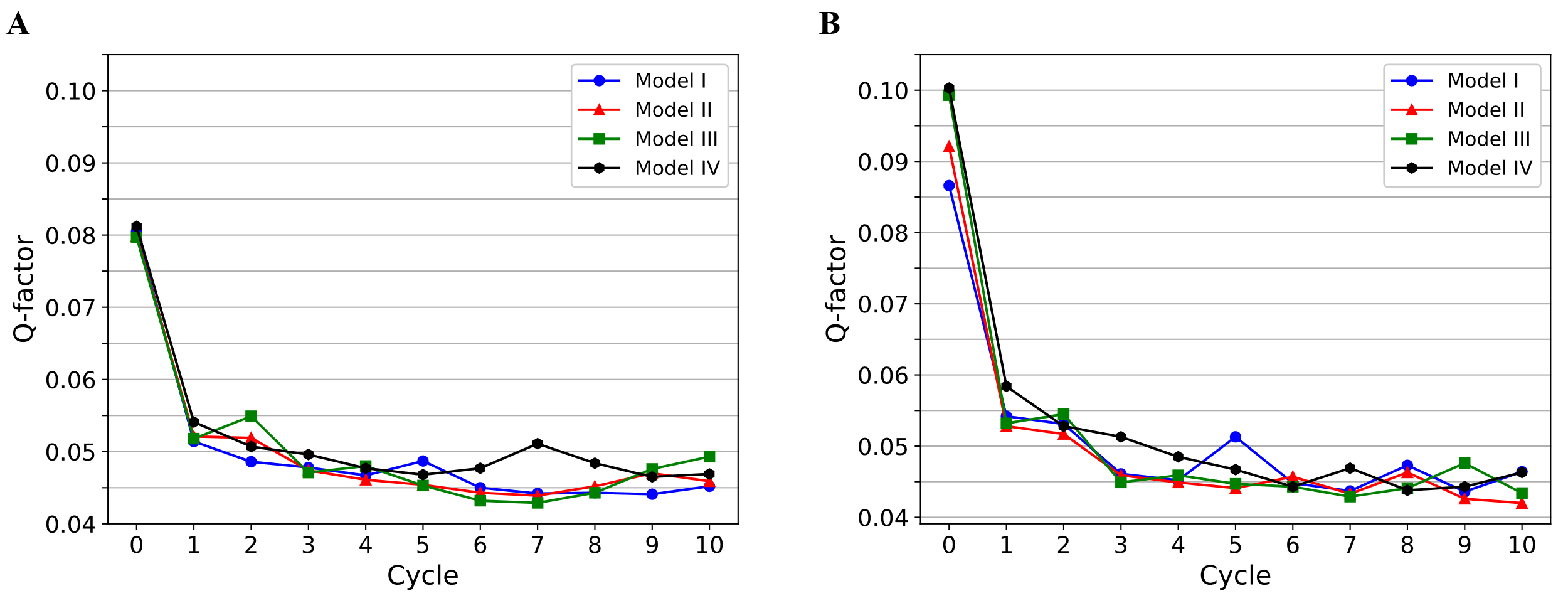
 **Fig S12** Q-factors calculated with Paramagpy during the YM_4_A iterative structure calculation for the M1 and M3 attachment sites. Each input model is depicted in a different series. **a** tagging site in module 3 (M3)S21C. **b** tagging site in module 1 (M1)Q18C.

**Table S13** Assignments of YM_4_A protein variants paramagnetically tagged at three different sites. Reported assignments refer to the total sequence, or to individual caps and modules. The average PCS slope was calculated by averaging the individual slopes connecting the dia- and paramagnetic species of each assigned peak.

|  |  | Start (cycle #0) | | | End (cycle #10) | | |
| --- | --- | --- | --- | --- | --- | --- | --- |
|  |  | Attachment site | | | Attachment site | | |
|  |  | **(A)S21C** | **(M3)S21C** | **(M1)Q18C** | **(A)S21C** | **(M3)S21C** | **(M1)Q18C** |
| Ax (10^-32^ m^3^) | Model I | 25.07 | 21.85 | 18.19 | 23.52 | 21.84 | 18.37 |
|  | Model II | 28.66 | 22.92 | 18.41 | 25.77 | 22.21 | 18.67 |
|  | Model III | 24.54 | 23.00 | 19.51 | 22.61 | 23.11 | 18.96 |
|  | Model IV | 20.25 | 21.71 | 18.24 | 19.78 | 21.84 | 17.81 |
| Rh (10^-32^ m^3^) | Model I | 9.58 | 4.33 | 7.84 | 11.34 | 5.22 | 3.12 |
|  | Model II | 10.00 | 5.44 | 8.51 | 10.90 | 5.95 | 3.34 |
|  | Model III | 9.87 | 4.91 | 8.60 | 10.56 | 5.18 | 3.20 |
|  | Model IV | 6.55 | 4.39 | 7.95 | 8.19 | 4.91 | 2.42 |
| Q-factor | Model I | 0.068 | 0.081 | 0.087 | 0.021 | 0.045 | 0.046 |
|  | Model II | 0.064 | 0.080 | 0.092 | 0.020 | 0.046 | 0.042 |
|  | Model III | 0.060 | 0.080 | 0.099 | 0.021 | 0.048 | 0.046 |
|  | Model IV | 0.065 | 0.081 | 0.100 | 0.021 | 0.047 | 0.046 |

**Table S14** Δχ-tensors details for the three paramagnetic tagging sites during the experimental refinement of YM_4_A. For each attachment site the axial (Ax) and rhombic (Rh) components are reported together with the corresponding Q-factor. At the start of the refinement (cycle #0, left three columns), a tensor is fitted for each of the four model structures I-IV, while at the end of the refinement (cycle #10, right three columns) the tensors are fitted for the refined structure.

**Table S15** Convergence of PCS-restrained YM_4_A refinements starting from three different seeds, used by CYANA to compute the starting conformer. For each input structure (see Materials and Methods), the calculation was performed as described in the Results with two additional seeds to evaluate the reproducibility and the RMSD between the resulting structures after 10 cycles of refinement along with the corresponding standard deviation is reported as convergence.

**Video S16** Individual phases of the simulated anneal (SA) of YM_4_A. The protein was annealed using the non-iterative approach as detailed in the Material and Methods, simulating PCS values from the three attachment sites with 100% assignments each and extracting snapshots every 200 MD steps. The relative plotting against the accuracy is shown in Fig 4. The SA is divided into four main phases, labelled in the top left corner. **phase 1** (red) the first snapshot after 200 MD steps is indicated as the starting point in a cartoon view, followed by the remaining 42 snapshots in ribbon view annealing. **phase 2** (orange) the last snapshot of phase 1 is indicated as the starting point in cartoon view followed by the remaining 42 snapshots in ribbon view annealing. The annealing movie is then repeated together with the last snapshot of phase 2 (yellow cartoon) to provide a better overview of the protein fold at the start and end of the phase. **phase 3** (yellow) the last snapshot of phase 2 is indicated as the starting point in cartoon view followed by the remaining 42 snapshots in ribbon view annealing. The annealing movie is then repeated together with the last snapshot of phase 3 (green cartoon) to provide a better overview of the protein fold at the start and end of the phase. **phase 4** (green) contains only two snapshots with extremely small structural changes, followed by a comparison between the result of the entire SA (green) and the input model structure (black) in cartoon view.


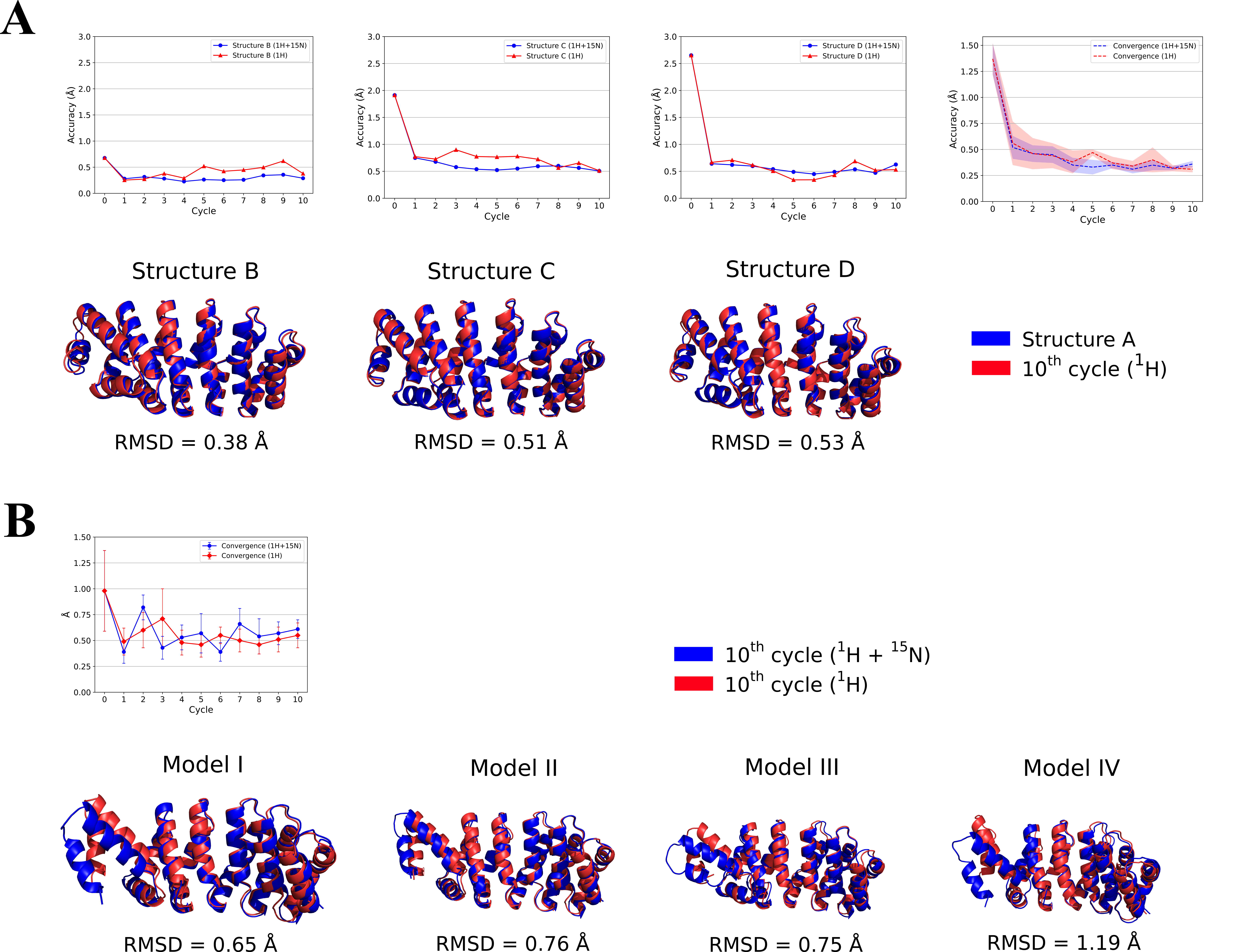


**Fig S17** Impact of backbone ^15^N PCS in the iterative refinement protocol. **a** simulations using the optimized iterative protocol with virtual PCS. The accuracy of the calculation is plotted versus the cycle number (top) when the calculation is performed in presence (blue) or absence (red) of ^15^N PCS. In all the simulations PCS values are computed from structure A (blue) but simulations start from different input structures, from left to right: structure B, structure C and structure D (see Materials and Methods). The resulting structure after 10 cycles of refinement is shown in red aligned with the target structure in blue (bottom). The RMSD after superposition of backbone heavy atoms of the entire sequence is indicated below. In the top-right corner, the RMSD between all three input structures during the refinement (convergence) is depicted by a dashed blue (presence of ^15^N PCS) or red (absence of ^15^N PCS) line with the shaded region indicating the standard deviation. **b** refinement using the optimized iterative protocol with experimental PCS. The RMSD between all four input structures during the refinement (convergence) is depicted by a blue (presence of ^15^N PCSs) or red (absence of ^15^N PCSs) line, calculated when excluding the Y cap and the first internal module, with error bars indicating the standard deviation. On the bottom, the resulting structure after 10 cycles of refinement is shown in blue (presence of ^15^N PCS) or red (absence of ^15^N PCS). The backbone heavy atom RMSD between the two structures, excluding the Y cap and the first module, is displayed below each alignment.
